# Systematic profiling of the chicken gut microbiome reveals dietary supplementation with antibiotics alters expression of multiple microbial pathways with minimal impact on community structure

**DOI:** 10.1186/s40168-022-01319-7

**Published:** 2022-08-15

**Authors:** Angela Zou, Kerry Nadeau, Xuejian Xiong, Pauline W. Wang, Julia K. Copeland, Jee Yeon Lee, James St. Pierre, Maxine Ty, Billy Taj, John H. Brumell, David S. Guttman, Shayan Sharif, Doug Korver, John Parkinson

**Affiliations:** 1 Department of Biochemistry, University of Toronto, Toronto, ON Canada; 2 Program in Molecular Medicine, Hospital for Sick Children, Peter Gilgan Center for Research and Learning, 686 Bay Street, Toronto, ON M5G 0A4 Canada; 3 Department of Agricultural, Food and Nutritional Science, University of Alberta, Edmonton, AB Canada; 4 Centre for the Analysis of Genome Evolution & Function, University of Toronto, 25 Willcocks St, Toronto, Ontario Canada; 5 Department of Molecular Genetics, University of Toronto, Toronto, ON Canada; 6 Program in Cell Biology, Hospital for Sick Children, Peter Gilgan Center for Research and Learning, 686 Bay Street, Toronto, ON Canada; 7 Institute of Medical Science, University of Toronto, Toronto, ON Canada; 8 SickKids IBD Centre, Hospital for Sick Children, Peter Gilgan Center for Research and Learning, 686 Bay Street, Toronto, ON Canada; 9 Department of Cell and Systems Biology, University of Toronto, Toronto, ON Canada; 10 Department of Pathobiology, Ontario Veterinary College, University of Guelph, Guelph, ON Canada

## Abstract

**Background:**

The emergence of antimicrobial resistance is a major threat to global health and has placed pressure on the livestock industry to eliminate the use of antibiotic growth promotants (AGPs) as feed additives. To mitigate their removal, efficacious alternatives are required. AGPs are thought to operate through modulating the gut microbiome to limit opportunities for colonization by pathogens, increase nutrient utilization, and reduce inflammation. However, little is known concerning the underlying mechanisms. Previous studies investigating the effects of AGPs on the poultry gut microbiome have largely focused on 16S rDNA surveys based on a single gastrointestinal (GI) site, diet, and/or timepoint, resulting in an inconsistent view of their impact on community composition.

**Methods:**

In this study, we perform a systematic investigation of both the composition and function of the chicken gut microbiome, in response to AGPs. Birds were raised under two different diets and AGP treatments, and 16S rDNA surveys applied to six GI sites sampled at three key timepoints of the poultry life cycle. Functional investigations were performed through metatranscriptomics analyses and metabolomics.

**Results:**

Our study reveals a more nuanced view of the impact of AGPs, dependent on age of bird, diet, and intestinal site sampled. Although AGPs have a limited impact on taxonomic abundances, they do appear to redefine influential taxa that may promote the exclusion of other taxa. Microbiome expression profiles further reveal a complex landscape in both the expression and taxonomic representation of multiple pathways including cell wall biogenesis, antimicrobial resistance, and several involved in energy, amino acid, and nucleotide metabolism. Many AGP-induced changes in metabolic enzyme expression likely serve to redirect metabolic flux with the potential to regulate bacterial growth or produce metabolites that impact the host.

**Conclusions:**

As alternative feed additives are developed to mimic the action of AGPs, our study highlights the need to ensure such alternatives result in functional changes that are consistent with site-, age-, and diet-associated taxa. The genes and pathways identified in this study are therefore expected to drive future studies, applying tools such as community-based metabolic modeling, focusing on the mechanistic impact of different dietary regimes on the microbiome. Consequently, the data generated in this study will be crucial for the development of next-generation feed additives targeting gut health and poultry production.

Video Abstract

**Supplementary Information:**

The online version contains supplementary material available at 10.1186/s40168-022-01319-7.

## Introduction

Over the past 70 years, the chicken industry has made remarkable gains in production efficiency largely driven by intensive breeding programs. Augmenting these breeding programs has been the use of sub-therapeutic doses of antibiotics (antibiotic growth promotants (AGPs)), added to chicken feed to enhance production efficiency [1]. AGPs are thought to operate through modulating the gut microbiome to limit opportunities for colonization by pathogens, increase nutrient utilization, and reduce inflammation [2–4]. However, due to global concerns over the association of AGPs with antimicrobial resistance (AMR) [5–7], there is increasing pressure to phase out their use; Europe banned their use in 2003, while the USA and Canada have recently implemented strategies to eliminate their use. Such bans are not without consequences [8]. In Europe, for example, the ban on AGPs has resulted in the increase of systemic infections [9], requiring greater application of therapeutic doses [10, 11]; a study of drug-free programs in Canada identified a reduction in production efficiency [12]. Consequently, to mitigate against the removal of AGPs, efficacious alternatives are urgently required. Given their role in modulating the microbiome, much attention has focused on the use of prebiotics and probiotics [13–15]. The key to identifying and developing appropriate formulations is understanding what impact AGPs have on the gut microbiome.

Previous studies of the chicken gut microbiome have typically used 16S rRNA sequence surveys to analyze community composition, i.e., which taxa are present and in what abundance [16–19]. Consequently, a major focus has been on community dynamics, with only limited functional insights. Prior to hatching, the chicken gut is considered sterile [20]; although bacterial DNA has been reported in eggs [21], live bacteria have yet to be recovered [22]. Development of the gut microbiome starts with an initial wave of colonization by aerobic and facultative anaerobes including *Escherichia coli*, lactobacilli, and *Streptococci* [23], after which obligate anaerobes including members of *Bacteroides*, *Bifidobacterium*, and *Clostridium* dominate. Although stable, these communities vary over the length of the gastrointestinal (GI) tract, with lactobacilli dominating the upper GI and Clostridiales dominating the lower GI [6, 7, 24, 25]. Helping define these community dynamics are alterations in diet, together with contributions from host genetics and other environmental factors such as geography and housing [26–31]. For example, chickens raised on different diets, such as corn or wheat, exhibit significant shifts in microbiota composition and function, reflecting differences in nutrient content [26, 27], while the use of reused (as opposed to fresh) litter has been shown to increase the relative abundance of *Faecalibacterium prausnitzii*, a notable butyrate producer [30]. Complementing these studies, several investigations have explored the impact of AGPs. For example, virginiamycin treatment reduced the relative abundance of lactobacilli [7, 32], although the effects were greatest in the small intestine. Consistent with this, avilamycin treatment has different effects on the ileum and cecal microbiome, increasing diversity in the former while reducing diversity in the latter [33]. However, in contrast, a study examining treatment with bacitracin methylene disalicylate found no significant impact on diversity in either ileal or cecal communities [34]. Together, these studies suggest that AGPs have a limited impact on microbiome structure that may be restricted to specific taxa at specific sites within the GI tract. However, due to their reliance on marker gene technologies such as 16S rRNA surveys, they yield only limited functional insights.

Here, we not only perform a comprehensive investigation of the role of diet, age, and AGP treatment on community structure across the chicken GI tract but also additionally apply whole microbiome RNA sequencing (metatranscriptomics), to reveal expressed functions and the taxa responsible [35–37]. Crucially, as we show here, the use of metatranscriptomics has the potential to reveal changes in microbiome function even in the absence of changes in community composition [38].

## Results

### Microbiome composition and diversity varies across GI site, diet, and age

**Fig. 1:**
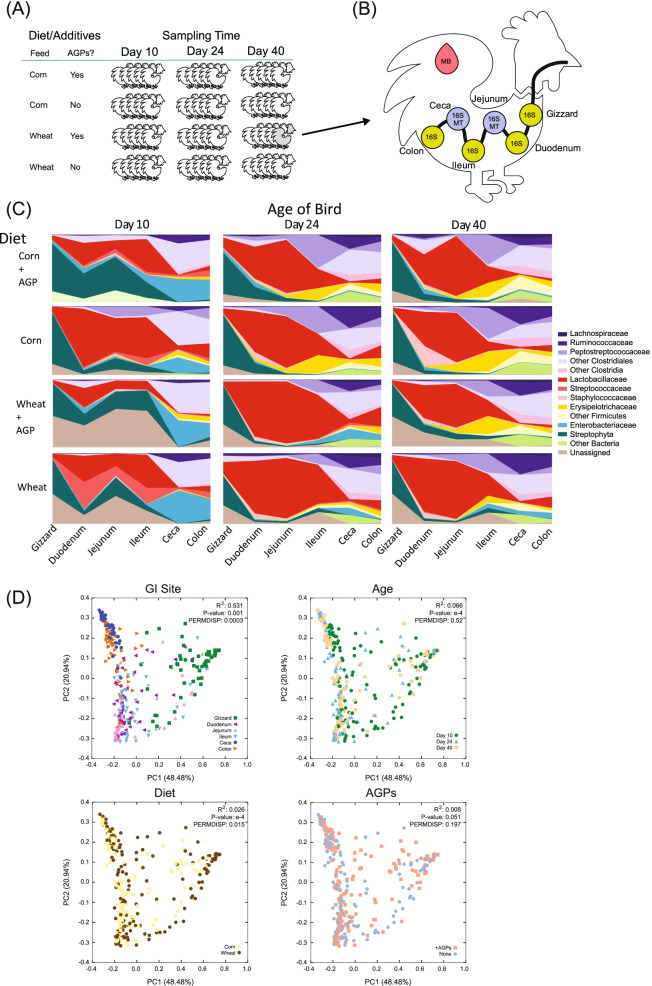
Overview of study design and summary of 16S rRNA data. **A** Birds are fed one of two diets (corn and wheat) in the presence and absence of AGPs to yield four conditions. Sets of five birds are harvested at days 10, 24, and 40. **B** From each bird, six intestinal sites were sampled for 16S rRNA analysis; two intestinal sites (jejunum and ceca) were sampled for metatranscriptomics and blood samples collected for metabolomics analysis. **C** Area plots showing major taxa identified in the six sampling sites of the chicken GI tract at days 10, 24, and 40 across 4 different treatments. Only taxa with an abundance > 1% were included. Note, relative to the older birds, the upper GI sites (gizzard, duodenum, and jejunum) of the 10-day-old birds featured a high proportion of reads mapping to *Streptophyta*, likely reflecting a reduced breakdown of the plant-based dietary components. **D** PCoA plots of weighted UniFrac distances for all samples and samples grouped by sampling sites, age, diet, and AGPs. For each plot, results from PERMANOVA and PERMDISP calculations are shown

To investigate the role of diet, age, GI site, and AGPs on microbiome structure and function, we raised 60 broilers (Ross 708, Aviagen) under four feeding regimes: corn based, wheat based, corn based with bacitracin methylene disalicylate (BMD; Zoetis Canada Inc., Kirkland, QC) and monensin (Coban, Elanco Canada Limited, Guelph, ON) supplementation, and wheat based with bacitracin and monensin supplementation (see “Methods”; Fig. 1). Consistent with commercial feed practices that optimize production, birds were initially fed a starter diet for 10 days post hatch, followed by a grower diet until day 24 and finally fed a finisher diet until day 40 post hatch (Supplemental Table 1). In line with these switches in diet, five birds from each regime were sacrificed at 10, 24, and 40 days post hatch. From each bird, blood samples were obtained for metabolomics analyses and the contents of six GI sites prepared for 16S rRNA survey sequencing. For birds sacrificed at days 24 and 40, we additionally performed whole microbiome RNASeq (metatranscriptomics) on jejunum and cecal samples. As expected, given the low number of replicates, no statistically significant differences in body weight were observed in pairwise comparisons between treatment groups (Supplemental Table 2).

Overall, we collected samples for 335 sites from 53 birds. From these samples, we generated 23,513,050 reads for 16S rRNA analysis of which 15,532,459 reads were used for analysis of OTUs (Fig. 1; Table 1; Supplemental Table 2). Read depth varied across samples, with the colon exhibiting the highest number of assigned reads (84,930 per sample) and the duodenum the lowest (9127 per sample). Across samples, we found that different body sites exhibited differences in community composition and complexity (Fig. 1; Tables 1 and 2; Supplemental Table 3). The colon and ceca samples had the greatest number of OTUs (51.5 and 52 per sample respectively), while the ileum, gizzard, and jejunum had the fewest (19.1, 20.4, and 21.1 per sample respectively). Unlike the jejunum and ileum, which tend to be dominated by members of *Lactobacillaceae*, duodenal samples contained additional members of the Staphylococcaeae family, together with unidentified members of the Clostridiales order absent from the jejunum or ileum. These additional taxa help drive the relative increase in diversity associated with the duodenum (Table 1; Supplemental Table 3).

**Table 1:** Overview of rRNA sequencing — average number of reads and OTUs generated for each site

GI site	Raw (pairs)	Merged (pairs)	Filtered (reads)	Final (reads)	Observed OTUs
Gizzard	78297.38	68914.39	56924.55	56770.07	20.41
Duodenum	20298.34	17733.02	9225.90	9127.33	40.34
Jejunum	41324.11	35743.49	25318.60	25236.29	21.06
Ileum	84388.45	73348.41	58219.43	57851.79	19.12
Ceca	77254.05	66116.41	47573.59	46057.88	51.95
Colon	121839.81	104843.98	86704.26	84930.36	51.45

**Table 2:** Influence of diet, AGPs, and combined treatment (“interaction” column) on microbiome structure as measured by PERMANOVA using weighted UniFrac distances

GI site	Time	Diet (Accounting for AGPs)		AGPs (Accounting for diet)		Interaction		
		R2	***p***-val	R2	***p***-val	R2	***p***-val	PERMDISP
**Gizzard**	**D10**	0.779	***	0.008	0.444	0.015	0.282	0.622
	**D24**	0.325	***	0.032	0.525	0.127	*	0.136
	**D40**	0.741	***	0.014	0.332	0.012	0.383	0.475
**Duodenum**	**D10**	0.121	*	0.227	**	0.052	0.229	0.16
	**D24**	0.105	0.169	0.024	0.856	0.023	0.863	0.988
	**D40**	0.064	0.218	0.125	*	0.037	0.637	**
**Jejunum**	**D10**	0.404	***	0.085	0.085	0.034	0.356	0.536
	**D24**	0.014	0.897	0.05	0.582	0.079	0.336	0.7332
	**D40**	0.053	0.386	0.049	0.442	0.035	0.67	0.363
**Ileum**	**D10**	0.297	***	0.202	**	0.098	*	0.547
	**D24**	0.187	**	0.024	0.808	0.074	0.218	0.81
	**D40**	0.308	***	0.025	0.609	0.029	0.529	0.182
**Ceca**	**D10**	0.13	0.052	0.04	0.486	0.085	0.141	0.941
	**D24**	0.249	**	0.066	0.229	0.043	0.461	**
	**D40**	0.105	**	0.136	***	0.077	*	0.066
**Colon**	**D10**	0.118	0.054	0.099	0.101	0.039	0.549	0.97
	**D24**	0.106	0.206	0.034	0.752	0.12	0.164	0.89
	**D40**	0.113	0.053	0.1	0.075	0.035	0.583	0.737

PERMDISP displays *p*-values as calculated through the PERMDISP test. Statistical significance is represented by the following: *p < 0.05; **p < 0.01; ***p < 0.001

As the GI tract transitions to a more anaerobic environment, we observed the replacement of *Lactobacillaceae* with Clostridiales as the dominant family in the ceca and colon. Indeed, in general, the taxonomic profiles of the ceca and colon were distinct from other sites, featuring obligate anaerobes from the Clostridia order (e.g., Ruminococcaceae and *Lachnospiraceae*), as well as many OTUs that could only be classified at the level of phylum (Firmicutes), class (Clostridia), or order (Clostridiales), resulting in the significant increase in diversity observed in these two sites (Supplemental Table 3). Linking the upper and lower GI, we found the ileum featured a distinctively high relative abundance of Erysipelotrichaceae particularly for birds fed a corn diet and sampled at days 24 and 40. Also noteworthy was a relatively high proportion of reads from day 10 samples obtained from the ceca and colon, mapping to Enterobacteriaceae, which decreases with the later timepoints (and almost absent in the birds fed the corn-based diets). Overall, these results are consistent with previous studies that suggest that GI site, diet, and age have a major impact on microbiome structure [16, 19, 25–27, 39, 40].

### Site of sampling and host age have greater impact on microbiome structure than diet and AGPs

To further examine the relative influence of GI site, diet, and age on microbiome structure, we performed a weighted UniFrac analysis (Fig. 1D) of all samples. This analysis reveals that site of sampling has the greatest impact on microbiome structure, followed by host age, with diet and AGPs having minimal relative impact. PCoA plots reveal a clear separation between samples collected from the gizzard and those from the ceca and colon, reflecting the significant physicochemical differences associated with these sites. Samples from the remaining three sites (duodenum, jejunum, and ileum) exhibit considerable overlap. Focusing on sampling age, we find that dispersion of samples depends on the site being sampled (Supplemental Fig. 1). For example, day 10 samples from the ceca and colon cluster separately from the more mature samples. Jejunal and ileal samples, particularly those associated with a wheat-based diet, also appear to delineate by age while those from the gizzard and duodenum do not. To examine the influence of diet and addition of AGPs on community structure, we grouped samples by age and sampling sites and performed PERMANOVA tests (Table 2). Our analysis reveals that diet had a greater impact on microbiome structure than AGPs, with more sites and timepoints exhibiting significant differences in microbiome structure. Furthermore, while the diet had a significant impact on microbiome structure across all three timepoints for the gizzard and ileum, the colon samples were unperturbed regardless of treatment.

Revisiting the diversity analyses (Supplemental Table 3) reveals a complex landscape of response to diet and AGPs that varies according to site and age of sampling. For example, under specific diet/AGP treatments, several sites respond with either an increase in diversity at day 24 relative to the other timepoints (e.g., gizzard/wheat and duodenum/wheat) or a decrease in diversity at day 24 relative to the other timepoints (e.g., duodenum/corn + AGPs and ileum/all treatments). Such nuance is otherwise lost when simply relying on changes in the relative abundance of different taxa (Fig. 1C).

Together, these findings highlight the complex relationships between diet, age, and AGPs, with microbial community structure across different sites in the GI tract. In the following section, we explore how these relationships might translate to changes in the relative abundance of specific taxa.

### Changes to specific taxonomic groups as a result of diet and AGPs

Representing one of the most diverse sites in the chicken GI tract, cecal microbiota have been shown to play a critical role in immune response [41] and growth performance [42]. We therefore examined how specific taxa in the ceca responded to changes in diet and AGP exposure. In initial analyses, we performed an ANOVA-like analysis (see “Methods”) to examine the impact of main effects (diet and AGPs) as well as their interaction on taxon abundance in day 24 and day 40 samples (Supplemental Table 4). Consistent with our previous PERMANOVA analyses on community structure (Table 2), changes in diet had a greater impact on the community than the application of AGPs. We also found a greater impact on the day 40 samples (37 taxa impacted by diet only, 12 taxa impacted by AGPs only, 4 taxa impacted by both diet and AGPs) relative to the day 24 samples (6 taxa impacted by diet only, 2 taxa impacted by AGPs only), likely reflecting the greater complexity associated with these samples. Furthermore, we found that diet and AGPs had a significant interaction effect on eight taxa in the day 40 samples (comprising four Clostridiales; two Enterobacteriaceae, and two uncharacterized Firmicutes) and only a single taxon in the day 24 samples (*Corynebacterineae* spp.). Given this complex landscape of the effects of age, diet, and AGPs on taxa, we next dissected these relationships by exploring each combination of diet and AGP in isolation.

**Fig. 2:**
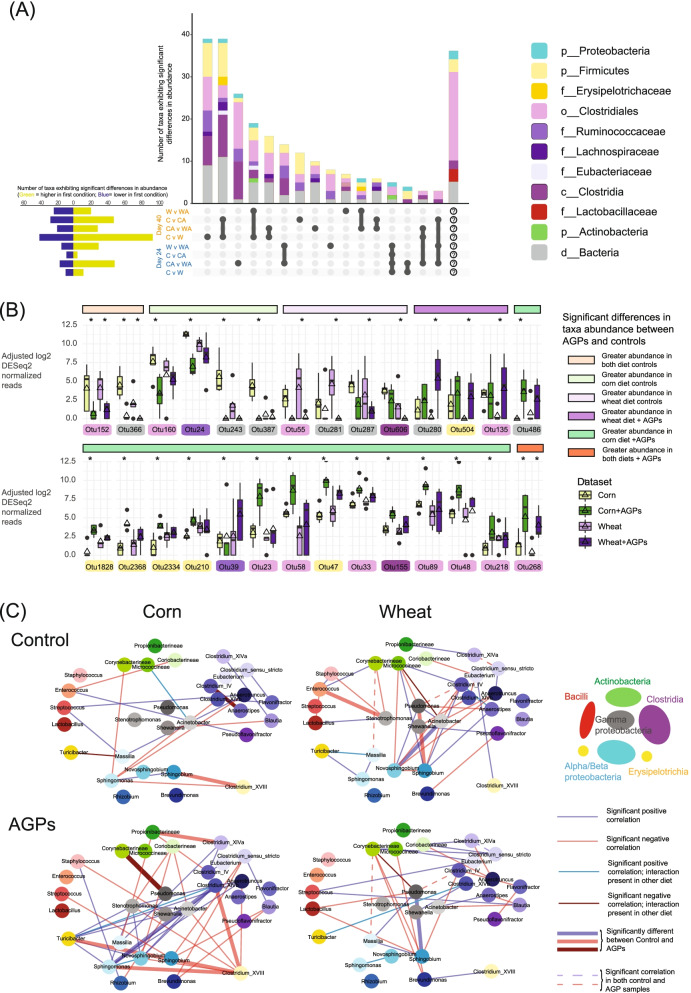
Microbial changes and interactions within the ceca. **A** Upset plot showing overlap in taxa exhibiting significant differential abundance across cecal comparisons between the four treatments over two timepoints (*W* = wheat; *WA* = wheat + AGPs; *C* = corn; *CA* = corn + AGPs). Only combinations with at least three taxa exhibiting significant changes are shown; remaining combinations are summarized in the last column. Note, to reflect the inability of 16S rRNA surveys to provide equal taxonomic resolution across all phyla and in line with previous studies [43], taxa are represented by a mix of taxonomic levels. **B** Box and whisker plots showing the 28 taxa that exhibit significant differences (as indicated by asterisks) in abundance due to AGPs (and not diet) in the day 40 cecal samples. **C** Co-occurrence networks generated with DGCA [44] for day 40 ceca samples. Each node represents a genus, shaded according to higher taxonomic levels (see inset). Links between genera indicate a significant correlation within that dataset

Applying DESeq2, we identified a total of 251 of the 1519 taxa associated with day 24 and day 40 cecal samples, exhibiting significant differences in abundance in the eight pairwise comparisons involving diet and AGPs (Supplemental Fig. 2; Supplemental Table 4). As before, most were associated with day 40 comparisons (187 taxa) with little overlap between day 24 and day 40 comparisons (25 taxa; Fig. 2A). Focusing on the day 40 samples, while there was relatively low concordance in responses to diet in the presence and absence of AGPs (i.e., comparisons involving corn v wheat — 134 taxa and corn + AGPs v wheat + AGPs — 48 taxa), of the 23 taxa that were significantly perturbed in both comparisons, 20 exhibited the same direction in response to diet, irrespective of AGPs. Since they were also unperturbed by the addition of AGPs when the diet did not change (i.e., comparisons involving corn v corn + AGPs and wheat v wheat + AGPs), these represent taxa that are sensitive to diet but unaffected by AGPs. Conversely, of the 28 taxa that responded only to AGP treatment in the day 40 samples (i.e., corn v corn + AGPs and/or wheat v wheat + AGPs), only 3 were common to both diets (Fig. 2B). Since these taxa also exhibited a similar direction of response to AGPs irrespective of diet, they represent taxa that are sensitive to AGPs but unaffected by diet. In general, however, consistent with the complexity observed in the diversity and main effects analysis, taxon responses to AGPs were dependent on diet. For example, in the day 40 samples, 43 taxa were perturbed only in the corn v wheat and corn v corn + AGPs comparisons, while 16 taxa were perturbed only in the corn v wheat and wheat v wheat + AGPs comparisons (Supplemental Fig. 2; Supplemental Table 4). The consistency in responses across conditions (e.g., higher abundance in corn relative to wheat or AGPs) suggests that AGPs may eliminate dietary-induced specificities associated with these taxa.

Analysis of the day 24 samples revealed that unlike the day 40 samples, more taxa were perturbed upon change in diet in the presence of AGPs (corn v wheat — 21 taxa; corn + AGPs v wheat + AGPs — 65 taxa). Also, unlike the day 40 samples, only half of the 10 taxa shared in these comparisons exhibited the same direction of response. This suggests that AGPs increase the sensitivity of the day 24 microbiome to dietary perturbation more than the day 40 microbiome. It is important to note, however, that these results may be confounded by the lower number of replicates associated with this timepoint for the two corn-based treatments (3 and 4 for the corn and corn + AGPs treatments, respectively).

In summary, at least for day 40 samples, more taxa were affected by diet than by AGPs. In addition, we identified taxa that were robust to AGPs but sensitive to a change in diet, as well as a smaller number of taxa that were robust to a change in diet but sensitive to AGPs. In the next section, we explore how these effects translate in changes in community organization.

### Co-occurrence networks reveal AGPs disrupt microbial community organization in the ceca

Previous analyses have shown that microbial communities in the chicken ceca may be organized through influential taxa that serve to promote the presence or absence of other taxa [31]. We were therefore interested in examining if AGPs might perturb interactions between taxa resulting in their mutual presence or absence. Due to their greater complexity, we focused our analyses on the day 40 cecal samples. Employing the Differential Gene Correlation Analysis R package (DGCA [44];), we constructed networks of interactions between genera for each diet/AGP combination. In addition to revealing interactions between taxa representing their mutual presence or exclusion in each sample type, we also identified interactions that were significantly altered in response to AGP exposure (Fig. 2C). Despite sharing the vast majority of genera, few interactions were shared between diets, and the addition of AGPs resulted in dramatic changes to their respective networks. Under a corn diet, AGPs promote a gain in correlations between the presence/absence of genera (44 v 18 significant correlations in the presence and absence of AGPs respectively). This gain was largely driven by a group of genera comprising positive correlations between *Acinetobacter*, *Novosphingobium*, *Stenotrophomonas*, *Turicibacter*, *Clostridirum* XIVb, *Sphingomonas*, and *Anaerotruncus*. Furthermore, two of these interactions (*Sphingomonas* and *Novosphingobium* and *Turicibacter* and *Clostridirum* XIVb) were common to both diets, suggesting they represent mutually supporting taxa, which together exert a significant influence on other taxa, such as the exclusion of *Clostridium* XVIII (associated with 3 of the 6 interactions that were significantly different between control and AGP samples under a corn diet). While no dramatic gain in correlations was observed for the wheat diet (40 v 31 significant correlations in the presence and absence of AGPs respectively), only 3 correlations were common to both treatments, again emphasizing a dramatic shift in community organization.

Based on our analyses of 16S rRNA datasets, relative to other factors such as diet and age, AGPs have only a limited impact on the composition of the chicken gut microbiome. However, the changes in community network structure observed under both diets suggest that AGPs mediate a key role in community organization, potentially defining influential taxa that may promote the exclusion of other taxa. The identification of these influencers and their ability to exclude potential pathogens may therefore offer alternatives to AGPs that promote poultry gut health. In the following sections, we explore how such organizational changes associated with diet and AGPs may also be related to functional changes in the microbiome.

### Taxonomic profiles derived from metatranscriptomics are similar but not identical to 16S rRNA surveys

To investigate functional changes within the chicken GI tract, we selectively deployed whole microbiome RNASeq (metatranscriptomics) to samples collected from birds at 24 and 40 days of age. We focused on two sites: jejunum (a key site of digestion and absorption) and the ceca (a major site for invasive enteric pathogens, even in apparently healthy birds). Across the 16 different combinations of diets, GI site, age, and AGP regime, we generated an average of ~35 million reads for each of 73 samples (Table 3; Supplemental Table 5). The resultant sequence datasets were parsed through our in-house metatranscriptomic pipeline (see “Methods”), identifying an average of 23.6 and 3 million reads that could be assigned to a microbial transcript for the cecal and jejunum samples respectively. Across all datasets, 184,692 unique bacterial transcripts were identified (Supplemental Table 6). The major source of sequence disparity between the two sites appears to be related to host contamination, with 8.3 and 28.8 million reads assigned to chicken transcripts for the cecal and jejunum samples respectively. While it is possible that jejunal gut epithelial cells may be more active than those in cecum, we expect that such a difference in host contamination largely reflects differences in the density of bacteria in the two locations [45].

**Table 3:** Summary of metatranscriptomics data generated from samples obtained from the jejunum and ceca

		GI site	
		Jejunum	Ceca
Samples		36	37
Read distribution (Average per sample)	Total reads	35,656,008	35,024,959
	Adaptor/low quality	882,679	1,220,289
	Dereplicated reads	6,819,170	14,545,294
	Host	28,831,231	8,329,729
	Diet associated	2,186,540	303,640
	rRNA	716,066	1,567,325
	Putative mRNA	3,039,403	23,603,976
	Reads mapped to known transcript	1,771,348	13,254,020
	Unique transcripts	49,354	184,279
	Unique enzymes	1,034	1,140

**Fig. 3:**
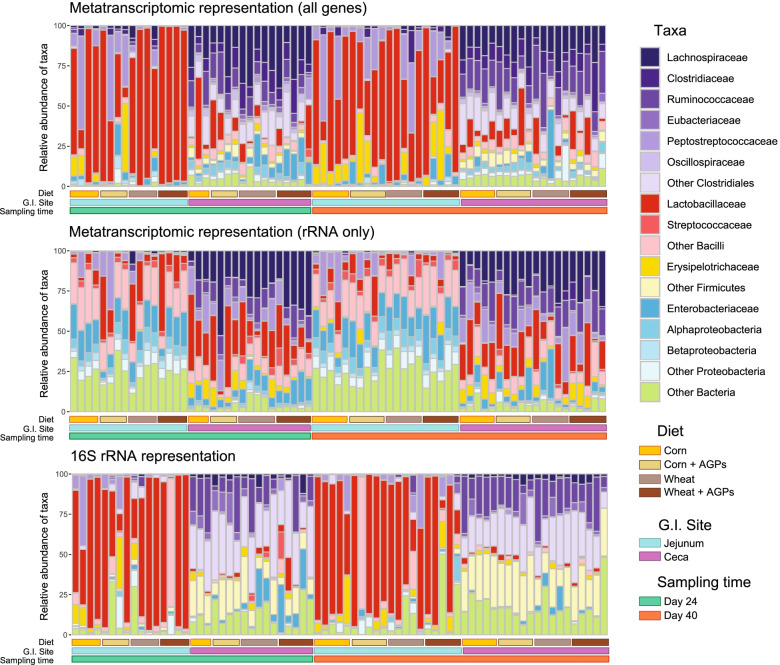
Comparison of taxon distributions for metatranscriptomic and 16S rRNA datasets. Taxonomic breakdown of sequences generated for each sample from the following: all metatranscriptomic reads mapping to a known gene/genome (top), metatranscriptomic reads mapping to rRNA sequences (middle), and mapped 16S rRNA reads (bottom)

Comparisons between the taxonomic representation of metatranscriptomic reads with those obtained from 16S rRNA surveys from the same samples reveal the two methodologies yield similar but not identical profiles (Fig. 3). For example, in the cecal samples, relative to metatranscriptomics, 16S surveys reveal an abundance of reads mapping to “Other Clostridiales,” “Other *Firmicutes*,” and “Other Bacteria,” suggesting taxa assigned to these groups have limited activity. Conversely, in the metatranscriptomic profiles of the cecal samples, we see an increase in the relative abundance of reads mapping to *Lachnospiraceae*, *Clostridiaceae*, *Lactobacillaceae*, Enterobacteriaceae, *Alphaproteobacteria*, and “Other Proteobacteria,” taxa that likely mediate highly active roles in the microbiome. Comparisons between jejunum samples exhibit similar discrepancies, with the day 40 samples being notable for an increase in transcripts from Erysipelotrichaceae. As for the 16S surveys, we find the jejunum samples are dominated by reads mapping to *Lactobacillaceae* transcripts. Interestingly, taxonomic profiles based on reads mapping rRNA in the metatranscriptomic dataset do not reflect those from either the complete set of metatranscriptomic assignments or those based on 16S surveys. This highlights taxonomic biases in the rRNA depletion kit used during preparation of the metatranscriptomic libraries and emphasizes why rRNA datasets derived from these libraries cannot be used to provide useful taxonomic readouts.

**Fig. 4:**
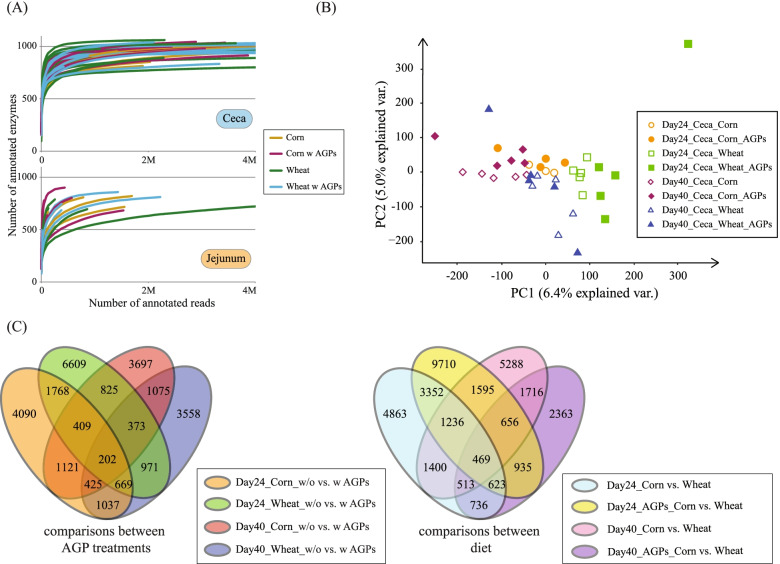
Overview of metatranscriptomic analysis. **A** Rarefaction analysis showing the number of enzymes detected (as defined by unique EC numbers) as a function of read depth for ceca and jejunum samples. **B** Principal component analysis (PCA) based on annotated microbial gene expression in ceca samples. Each node represents an individual ceca sample (see inset key for type of sample). **C** Overlap of significantly differentially expressed genes across ceca samples, comparing AGP treatments (left) and diet (right)

Beyond taxonomic representation, we find that the cecal samples are associated with over three times more unique microbial genes than the jejunum samples (184,279 v 49,354, respectively). However, the cecal samples are only associated with ~100 more unique enzymes. Rarefaction analyses suggest that while the sequencing depth associated with cecal samples was sufficient to recover most enzymatic activities, many jejunum samples do not appear saturated in terms of sequence coverage (Fig. 4A). Consequently, we focus our subsequent analyses on cecal samples. Principal component analysis based on gene expression of the cecal samples reveal the first two components are able to separate samples on the basis of diet and, at least for wheat samples, day of sampling (Fig. 4B). Furthermore, these components also separate two types of AGP samples (day 24 wheat and day 40 corn), suggesting that at least under these combinations of diet:day of sampling, the addition of AGPs impacts microbial gene expression. To examine diet and AGP effects further, we performed differential expression analysis, using DESeq2 [46, 47] within an ANOVA-like framework similar to that performed on taxa abundances (see “Methods”; Supplemental Table 7). Consistent with our previous analyses, changes in diet had a greater impact on the community than the application of AGPs, and day 40 samples (3422 genes impacted by diet; 866 transcripts impacted by AGPs) were more affected than day 24 samples (1218 transcripts impacted by diet; 23 genes impacted by AGPs). Interestingly, we identified only 30 transcripts in the day 40 samples (and none in the day 24 samples) as being subject to diet by antibiotic interactions. This may reflect both the relatively low number of samples used in this study and the large number of microbial genes identified, which together limit our ability to identify genes subject to diet by AGP interactions. Therefore, to further dissect these relationships further and identify the types of genes and functions impacted by diet and AGPs, we performed pairwise comparisons of gene expression differences for each combination of diet and AGP in isolation.

From our pairwise comparisons of AGP treatments, we identified 26,829 and 1059 microbial transcripts that exhibited significant differential expression for cecal samples and jejunum samples respectively (Fig. 4C). For the pairwise comparisons of diets, we identified 35,461 and 990 microbial transcripts that exhibited significant differential expression for cecal samples and jejunum samples respectively. Although most of these genes were unique to a specific pairwise comparison, we identified core sets of 202 and 469 microbial transcripts that exhibited significant differential expression across all AGP and dietary comparisons respectively (Supplemental Table 8). Gene set enrichment analyses (GSEA) reveal these core sets are associated with several metabolic pathways as defined by the Kyoto Encyclopedia of Genes and Genomes (KEGG; Supplemental Table 9). For the core set of dietary comparisons, we identified several pathways involved in nucleotide metabolism (purine and pyrimidine metabolism), amino acid biosynthesis (alanine, aspartate and glutamate metabolism, and arginine biosynthesis) and the production of energy (glyoxylate and dicarboxylate metabolism; pentose phosphate pathway and glycolysis). This likely reflects transcriptional responses to different nutrient environments. For the AGP comparisons, in addition to the two amino acid pathways identified in the diet comparisons, we also noted an enrichment of the purine pathway. This latter pathway is of interest as previous studies have shown that cellular stress associated with antibiotics results in a depletion of purine nucleotides [48].

Having identified metabolic pathways associated with core sets of genes associated with changes in diet or administration of AGPs, in the following section, we extend these analyses to further dissect the impact of AGPs, diet, and age on the expression of metabolic enzymes and pathways encoded by the microbiome.

### AGPs alter the expression of multiple metabolic pathways

**Fig. 5:**
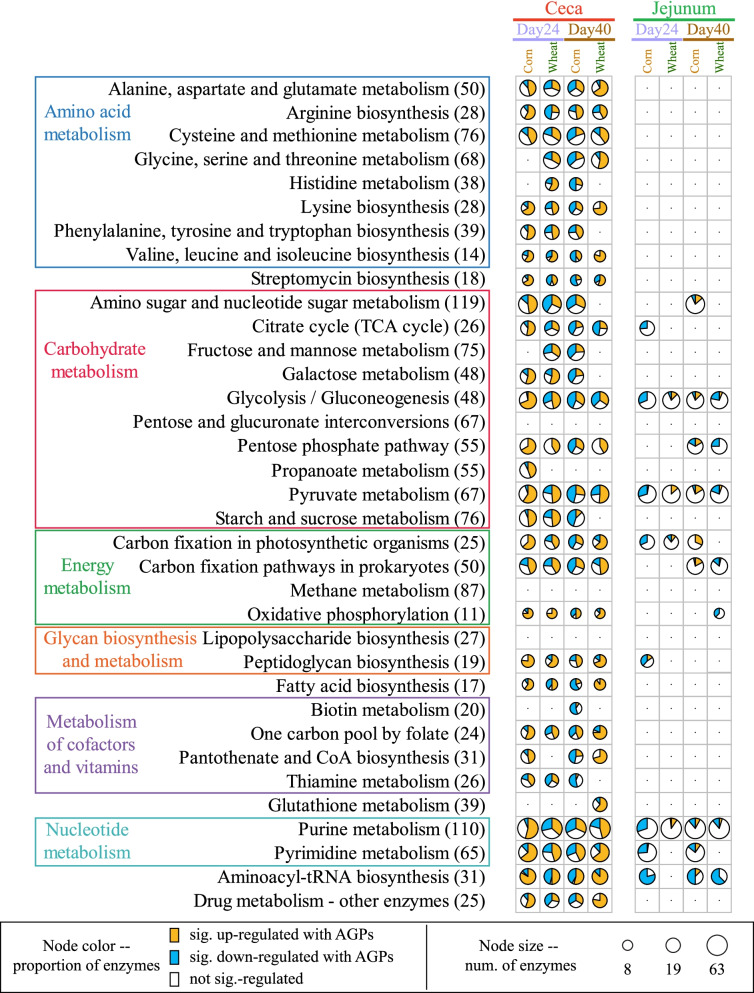
Metabolic pathways significantly enriched with enzymes exhibiting significant changes in abundance in the presence and absence of AGP. Thirty-two pathways as defined by KEGG were enriched in enzymes exhibiting significant changes in expression in either ceca or jejunum samples, in the presence or absence of AGPs. Each pie chart shows the proportion of enzymes that were significantly up- (orange) or down-(blue) regulated in the presence of AGPs. White sectors indicate enzymes that were identified in the pathway but which did not exhibit significant differential expression

Focusing on the set of 26,829 transcripts that exhibited significant differences in expression across the four pairwise comparisons involving AGPs in the ceca (i.e., day 24, the presence and absence of AGPs for corn- and wheat-based diets; day 40, the presence and absence of AGPs for corn- and wheat-based diets), we identified 744 unique enzymes. Placing these in the context of KEGG-defined metabolic pathways and applying GSEA identified 32 pathways significantly enriched in these enzymes, many featuring complex patterns of response to diet and/or AGPs (Fig. 5). Of these, 20 pathways exhibited significant differences across all four comparisons. We also identified several pathways restricted to specific comparisons. For example, five pathways (phenylalanine, tyrosine and tryptophan biosynthesis, amino sugar and nucleotide sugar metabolism, galactose metabolism, starch and sucrose metabolism, and thiamine metabolism) exhibited significant changes in pathway expression across all comparisons with the exception of day 40 wheat-based samples. These and other pathways restricted to specific comparisons are likely related to changes in other feed ingredients associated with the grower and finisher diets; relative to the finisher diets, the grower diets contain a higher percentage of soybean meal at the expense of corn/wheat, canola meal, and vegetable fat (Supplemental Table 1). From the profiles of changes in enzyme expression observed in the four pairwise comparisons (Supplemental Table 10), we found that the corn-based diets exhibit fewer upregulated enzymes in the presence of AGPs than the wheat-based samples at 24 days (340 v 592 enzymes respectively). However, this pattern appears reversed for the 40-day samples (556 v 310 enzymes respectively). Interestingly, differentially expressed enzymes associated with the pentose phosphate pathway were all upregulated in the presence of AGPs, with the exception of samples derived from birds fed a corn diet at day 40, which also exhibited some enzymes that were downregulated. This diet-sensitive pattern suggests that AGPs may interfere with regular pathways microbiota rely on for energy production.

Related comparisons of the 35,461 transcripts exhibiting significant differential expression between diets (i.e., day 24, corn v wheat in the presence and absence of AGPs; day 40, corn v wheat in the presence and absence of AGPs) revealed 1141 unique enzymes (Supplemental Fig. 3; Supplemental Table 10) enriched in 33 KEGG-defined pathways. Of these, 22 pathways exhibited significant differences across all four comparisons (day 24, corn v wheat in the presence and absence of AGPs; day 40, corn v wheat in the presence and absence of AGPs). As for the comparisons between AGPs and controls, we identified several pathways restricted to specific comparisons. For example, four pathways (galactose metabolism, pentose and glucuronate interconversions, starch and sucrose metabolism, and thiamine metabolism) were restricted to the 2-day 24 comparisons. Conversely, pantothenate and CoA biosynthesis were restricted to day 40 comparisons. As noted above, the restriction of enzyme expression patterns to specific pairwise comparisons is likely a reflection of changes in diets used to raise the birds. Profiles of enzyme expression patterns in these dietary comparisons revealed that at 24 days, fewer enzymes are significantly upregulated under a wheat-based diet relative to a corn-based diet in the absence of AGPs (186 v 522 enzymes in the absence and presence of AGPs respectively; Supplemental Fig. 3; Supplemental Table 10). As before, this pattern was reversed for the day 40 samples (522 v 262 enzymes in the absence and presence of AGPs respectively). Consistent with the analysis performed for all transcripts, principal component analysis (PCA) of enzyme expression profiles (Supplemental Fig. 4) reveals samples from day 24 birds fed wheat with AGPs, cluster separately from the other samples. This again highlights complex interactions between diet and AGP use on microbial gene expression.

For the jejunum samples, consistent with transcript expression, we identified fewer enzymes exhibiting differential expression across samples and consequently fewer metabolic pathways enriched in these enzymes (Fig. 5). Notable pathways exhibiting consistent differential responses include glycolysis, pyruvate metabolism, and purine metabolism. For both cecal and jejunum samples, these pathways were also among those exhibiting the highest levels of expression (Supplemental Fig. 5). Conversely, pathways involved in amino acid metabolism, lipopolysaccharide biosynthesis, fatty acid biosynthesis, and co-factor and vitamin metabolism exhibited the lowest levels of expression.

Our findings that AGPs impact the expression of many pathways involved in carbohydrate metabolism and energy production are in line with a recent study of the murine gut microbiome, which found that antibiotic exposure, particularly in the presence of glucose and polysaccharides, alters metabolic pathway expression [49].

### Diet and AGPs alter enzyme expression patterns in pathways involving the production of energy

**Fig. 6:**
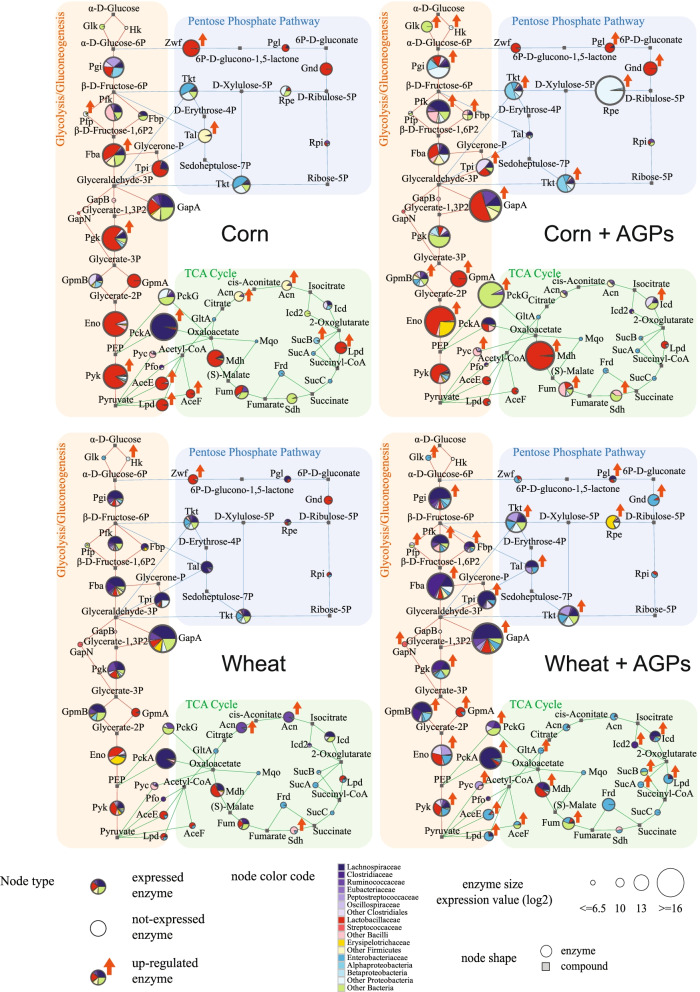
Taxonomic contributions to gene expression for enzymes involved in energy production. The expression of enzymes involved in glycolysis/gluconeogenesis, pentose phosphate, and tricarboxylic acid (TCA) cycle pathways is indicated for cecal samples obtained from day 24. Each pie chart represents the taxonomic contributions of enzyme expression (see key for color code). The size of pie charts indicates the average expression value (with log2 transform) of enzymes across all samples analyzed for each condition. Red arrows indicate enzymes that are significantly upregulated in comparisons involving the presence/absence of AGPs. Enzyme abbreviations are listed in Supplemental Table 11

Given their interconnected roles in the production of energy, relatively high expression, and response to dietary/AGP treatments, we examined changes in the patterns of expression of enzymes involved in the energy production pathways (glycolysis, the pentose phosphate pathway, and the TCA cycle; Supplemental Table 11). Comparing the samples collected at 24 days (Fig. 6), *Lactobacillaceae* dominate enzyme expression in glycolysis under a corn-based diet, while *Lachnospiraceae*, *Clostridiaceae*, and Enterobacteriaceae dominate under a wheat-based diet. This is consistent with the change in taxonomic profiles associated with all transcripts relative to the 16S rRNA datasets, emphasizing that these two taxa are among the more active in the community (Fig. 3). At the same time, it is interesting to note the dominance of the *Lactobacillaceae* in the corn diet, despite both diets exhibiting similar relative abundances of *Lachnospiraceae*. Such taxonomic differences in glycolytic enzyme representation were absent in the samples collected at 40 days (Supplemental Fig. 6). Similarly, while AGPs significantly increase the expression of multiple enzymes in glycolysis in the day 24 samples (12 v 7: corn v corn + AGPs; 19 v 1: wheat v wheat + AGPs), day 40 samples exhibited fewer differences in the number of glycolytic enzymes with increased expression (10 v 9: corn v corn + AGPs; 11 v 10: wheat v wheat + AGPs).

Comparisons of enzyme expression between pathways reveal relatively high levels of expression in glycolysis compared to the pentose phosphate pathway and the TCA cycle, across all treatments. While it is important to note that enzyme expression does not directly correlate with pathway flux, these findings nonetheless suggest that glycolysis is a major route for energy production. Relatively few enzymes appear consistently differentially regulated across all conditions. Examples include phosphoglycerate mutase (GpmA, EC 5.4.2.11), which appears upregulated in the presence of AGPs, while glucose-6-phosphate isomerase (Pgl, EC 5.3.1.9), phosphogluconate dehydrogenase (Gnd, EC 1.1.1.44), and ribulose-5-phosphate 3-epimerase (Rpe, EC 5.1.3.1), members of the pentose phosphate pathway, are also consistently upregulated in the presence of AGPs. We speculate that this may be related to the production of ribose-5-phosphate, an important precursor for purine metabolism; as noted above, cellular stress associated with antibiotics results in a depletion of purine nucleotides [48]. The downregulation of components of the pyruvate dehydrogenase complex (AceE, AceF, and Lpd), responsible for the production of acetyl-CoA, in the presence of AGPs in day 40 samples, as well as the corn sample from day 24, suggests a potential shift in the flux of metabolites feeding into the TCA cycle.

In terms of taxonomy, many enzymes exhibit similar representation in their expression in the presence and absence of AGPs (e.g., glyceraldehyde-3-phosphate dehydrogenase (GapA, EC 1.2.1.12), transketolase (Tkt, EC 2.2.1.1), fructose-bisphosphate aldolase (Fba, EC 4.1.2.13), phosphoglycerate mutase (GpmB, EC 5.4.2.12), and 6-phosphofructokinase (Pfk, EC 2.7.1.11)). Notably, the latter lacks representation by *Lactobacillaceae* in corn-based samples. Given broad representation of *Lactobacillaceae* across glycolysis in these samples, we suggest that transcripts assigned to the enzyme that are classified as “Other *Bacilli*” lack resolution to be classified at the higher taxonomic level of *Lactobacillaceae*.

In summary, these investigations reveal AGPs have a dramatic impact on the expression of enzymes in core energy production pathways, with samples from day 24 exhibiting an overall increase in expression. That similar changes were not observed in the day 40 samples may reflect differences in response to the grower and finisher diets used prior to collection of these samples.

### Diet and AGPs alter enzyme expression patterns in purine metabolism

Given the enrichment of significantly differentially expressed enzymes in the purine pathway and its previous association with antibiotic usage, we next examined gene expression in the context of the complementary, de novo biosynthetic and salvage pathways (Supplemental Figs. 7 and 8; Supplemental Table 11). Focusing on the day 24 ceca samples (Supplemental Fig. 7), as for the energy production pathways highlighted above, we again observe relatively high representation of *Lactobacillaceae* and *Lachnospiraceae* in the corn- and wheat-based diets respectively, although such dominance is restricted to the salvage pathway for the corn-based samples. Furthermore, representation by these two taxa is more limited in the samples collected at 40 days (Supplemental Fig. 8). More consistently, for both timepoints, we observe a greater diversity in taxonomic representation for the four enzymes involved in the interconversions involving guanosine, xanthosine, and inosine (5′-nucleotidase (SurE, EC 3.1.3.5), xanthine phosphoribosyltransferase (Xpt, EC 2.4.2.22), purine-nucleoside phosphorylase (PunA, EC 2.4.2.1), and hypoxanthine phosphoribosyltransferase (HprT, EC 2.4.2.8)). With the addition of AGPs, we see notable differences in the taxonomic representation of enzymes involved in the salvage pathway. For example, corn samples exhibit reduced representation by *Lactobacillaceae*. Across the entire pathway, we found more enzymes significantly upregulated in the presence of AGPs for three of the four comparisons (19 v 14 and 31 v 5 for corn- and wheat-based samples collected at day 24; 26 v 10 for corn-based samples collected at day 40). The single exception was for wheat samples collected at day 40 which displayed similar numbers of significantly upregulated enzymes in the presence and absence of AGPs (18 v 18). We observed fewer taxonomic perturbations associated with the de novo pathway expression profiles, with similar taxonomic contributions for multiple genes. Intriguingly, the expression of some enzymes was dominated by a single taxon. Most notably, across all samples, *Lactobacillaceae* dominate expression of deoxyguanosine kinase (Dgk, EC 2.7.1.113), while other enzymes exhibit such taxon domination only under specific conditions (e.g., adenylate kinase — Adk: EC 2.7.4.3— represented by Erysipelotrichaceae in day 24 Corn and 5′-nucleotidase — SurE: EC 3.1.3.5 — represented by Oscillospiraceae in day 24 wheat + AGPs.

Overall, these results highlight complex relationships between the expression (and taxa responsible) of enzymes involved in purine biosynthesis and AGP treatment that may reflect a response to cellular stress and depletion of purine nucleotides [48]. Further interpretation of these changes would benefit from the application of metabolic modeling approaches, capable of predicting metabolic flux and growth for individual taxa [50, 51].

### Age-dependent metabolic changes disappear in the presence of antibiotics

**Fig. 7:**
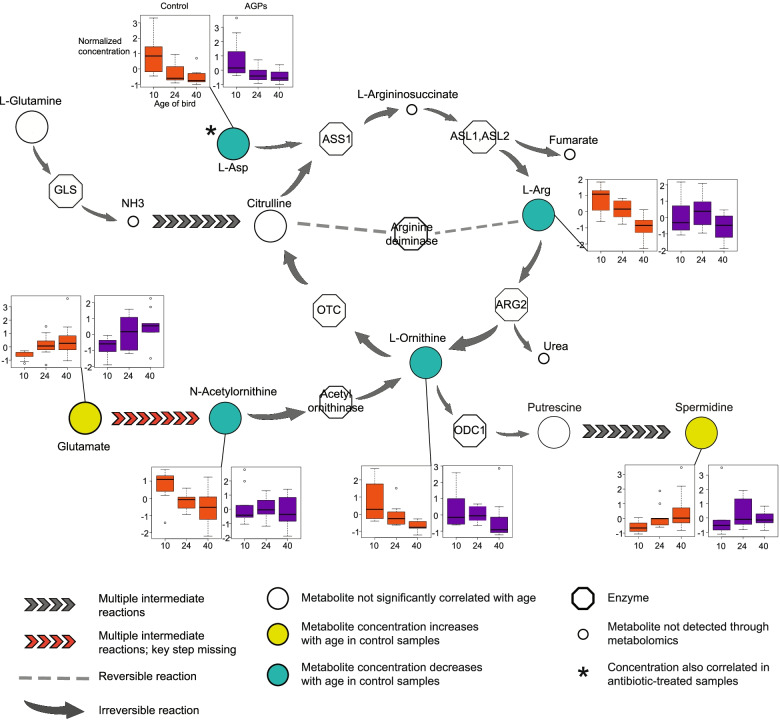
Metabolites associated with the urea cycle do not decrease with age in the presence of AGPs. The main graphic shows a section of the urea cycle indicating metabolites with concentrations that change with age. Bar graphs indicate normalized metabolite concentrations at each of the three timepoints for samples obtained from control and AGP-treated birds. With the exception of L-Asp, age-related changes in metabolite concentrations are associated only with control samples. Note chickens are thought to lack N-acetylglutamate synthase, a key enzyme in the conversion of glutamate to N-acetylornithine [52]

In an attempt to shed light on the downstream consequences of changes in metabolic pathway expression, we applied targeted metabolomics to serum samples collected from all 60 birds used in the study. The resultant profiles capture 139 metabolites, covering a variety of carbohydrates, fatty acids, and amino acids. To examine the impact of diet and AGPs, as well as their interaction, we performed a permutation-based factorial ANOVA for each metabolite, stratifying by age. We identified no main or interaction effects on any metabolite at any of the three timepoints. However, principal component analysis (PCA) found that in the absence of AGPs, these profiles segregated according to age of bird (Supplemental Fig. 9). This suggests that the birds exhibit age-related changes in their metabolism, potentially due, at least in part, to the various diets used in the poultry life cycle. Interestingly, this age effect is lost in birds fed AGPs. To investigate this effect further, we identified metabolites correlating with age in both control and AGP-treated samples. Consistent with the PCA, 55 and 27 metabolites were found to correlate with age in control and AGP-treated samples respectively (Supplemental Table 12). Among the 55 metabolites identified as correlating with age in control (and not AGP-treated) samples, four of the six metabolites associated with the urea cycle significantly decreased with bird age (Fig. 7). Despite possessing many of the enzymes associated with the urea cycle, this pathway is considered nonfunctional in chickens, potentially due to the lack of N-acetylglutamate synthase (NAGS), and may therefore provide some alternative function [52]. As NAGS converts glutamate to N-acetyl glutamate, its absence in the chicken genome may in part explain the age-associated increase in glutamate that appears contrary to other metabolites in this pathway.

These results indicate that the influence of AGPs extends beyond microbial metabolism to directly impact metabolites in the host. Further studies are needed to dissect the mechanisms involved and the downstream consequences for the host.

### AGP supplementation increases expression of microbial genes involved in cell wall biogenesis

Since the two AGPs used in this study both target the cell wall (monensin targets cell membranes and disrupts the ion gradients required for nutrient transport and the generation of the proton motive force, while bacitracin interferes with the biosynthesis of cell walls), we were interested in transcriptional responses of genes involved in cell wall biogenesis. To explore this, we integrated our metatranscriptomic data into a high-quality map of protein-protein interactions for gene products involved in cell wall biogenesis and cell division previously generated for *E. coli* [53].

**Fig. 8:**
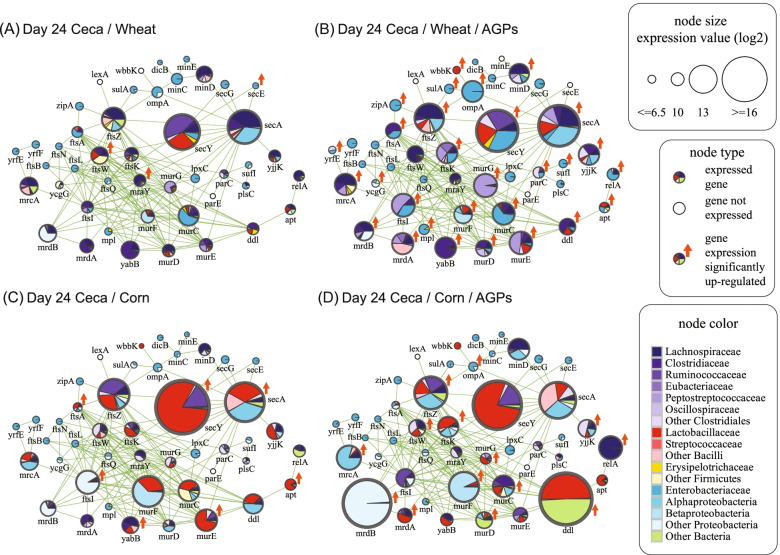
Taxonomic contributions to gene expression profiles for proteins involved in cell wall biogenesis for ceca samples collected at day 24. Each node in the network indicates groups of orthologs corresponding to a specific *E. coli* gene (as indicated) involved in cell wall biogenesis. Links between nodes indicate a functional interaction as previously defined [53]. Size of the node indicates the relative expression of genes associated with each set of orthologs, with sector colors indicating the taxonomic contribution to gene expression (see key for color code). Red arrows indicate sets of orthologs that are significantly upregulated in comparisons involving the presence/absence of AGPs

Comparing between the 24-day cecal samples, we observed a consistent increase in the expression of genes involved in cell wall biogenesis in the presence of AGPs, with 29 and 14 (of 45 total) gene families upregulated under wheat and corn diets respectively, compared with only 3 and 7 genes families exhibiting downregulation (Fig. 8). Notably, 13 of the upregulated gene families were common to both diets, including the outer membrane protein, ompA, the cell division genes ftsZ and ftsK, and multiple genes involved in peptidoglycan biosynthesis (mrcA, mrdA, mrdB, murC, murD, murF, murG, ddl). Compared to changes in diet, the addition of AGPs reveals subtle differences in the taxonomic representation of gene expression. For example, under a wheat diet, the increase in expression of homologs of secA, ftsA, and murE associated with AGP treatment is driven at least in part by *Lactobacillaceae*. Similarly, under a corn diet, the increase in the expression of mrcA, mrdB, and murF homologs associated with AGP-treated samples is driven by various clades of Proteobacteria. Consistent with previous analyses of mouse ceca and cow rumen microbiomes [36], we found that ftsZ, secA, and secY were among the most highly expressed genes, likely reflecting their essential roles and high conservation. Interestingly, under a corn diet, the addition of AGPs resulted in a dramatic increase in the expression of mrdB and ddl, driven by reads assigned to the genus *Helicobacter* for the former and by reads assigned to *Lactobacillus* and *Corynebacterium* for the latter. In addition to predicting a potential mechanism by which these taxa respond to AGP exposure, these findings again highlight the complex interplay between diet, AGPs, and gene expression.

Focusing on cecal samples from 40-day-old chickens, we observed a similar pattern of increased expression of genes involved in cell wall biogenesis with AGPs (Supplemental Fig. 10). As for the 24-day samples, various clades of proteobacteria appear to be associated with increases in the expression of several genes including homologs of murF, yjjK, ftsZ, and ftsK. However, for the samples obtained from the wheat diet, the addition of AGPs did not reveal a consistent pattern of response. For example, while the expression of secA homologs increased due to greater representation by *Lactobacillaceae*, the expression of homologs of mrcA, ftsI, and ftsK exhibited decreased expression, particularly for *Lactobacillaceae*.

As with overall taxonomic contributions to gene expression (Fig. 3), cell wall biogenesis gene expression in jejunal samples were largely associated with a more limited set of taxa including Erysipelotrichaceae, *Lactobacillaceae*, and Peptostreptococcaceae. Furthermore, unlike the 24-day-old cecal samples, both 24- and 40-day jejunal samples did not exhibit any consistent changes in patterns of expression of cell wall biogenesis genes (Supplemental Fig. 11). Together, these findings suggest that the greater influence of AGPs on the cecal microbiome may reflect its slower passage rate relative to the other GI sites [26] resulting in a longer exposure to the effects of AGPs.

### AGP supplementation increases expression of antimicrobial resistance genes

**Fig. 9:**
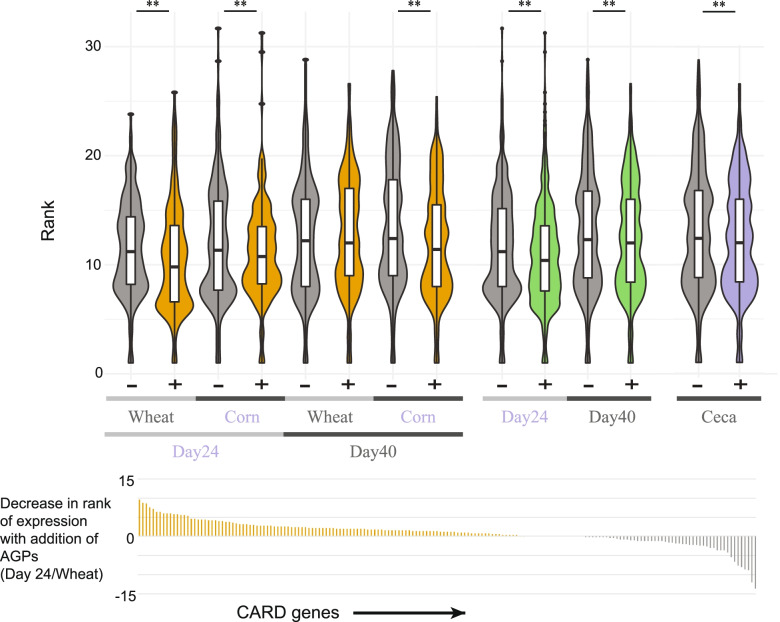
Antimicrobial resistance gene expression increases with AGP supplementation. Top panel: violin plots showing distribution of ranks of the expression values of 179 CARD genes for each sample type (1st (highest) rank = highest expression across all cecal samples). With the exception of day 40 wheat samples, CARD genes exhibit significantly higher rankings of expression in samples from birds fed AGPs relative to controls (** = *p* < 0.01 two-sample paired *t*-test). Lower panel: change in ranking of 179 CARD genes (ranked across all cecal samples) in day 24 wheat samples upon supplementation with AGPs

There have been numerous studies linking the use of antibiotics with an increase in antimicrobial resistance (AMR). We therefore examined if AGP supplementation resulted in an increase in the expression of genes associated with AMR. For each sample, we used the Resistance Gene Identifier (RGI) tool to predict their resistome based on homology and SNP models derived from the Comprehensive Antibiotic Resistance Database (CARD) [54]. Across all cecal samples, we predict 179 transcripts associated with five categories of resistance mechanism: antibiotic efflux, antibiotic target protection, antibiotic inactivation, antibiotic target alteration, and reduced permeability to antibiotic (Supplemental Table 13). To reduce the impact of individual AMR genes exhibiting high expression, we ranked the expression of each gene and analyzed the shift in ranking in the presence and absence of AGPs (Fig. 9). With the exception of birds fed a wheat-based diet and sampled at day 40, the addition of AGPs significantly increased the relative expression of AMR gene expression. Interestingly ~50% of AMR genes detected were assigned to homologs from two taxa: *Escherichia coli* IAI39 (41 genes) and *E. coli* O157:H7 str. SS17 (48 genes). Expression of these genes suggests these two taxa are mutually exclusive, with individual metatranscriptome datasets associated with only one of the two taxa. Noteworthy, AMR expression in jejunal samples was dominated with representatives from *E. coli* O157:H7 str. SS17 but not *Escherichia coli* IAI39 (Supplemental Table 14). Together, these findings reveal AGPs to exert effects on additional microbial pathways beyond metabolism, with the potential to directly impact cell growth and division.

## Discussion

In this study, we performed a systematic survey of microbial communities in the gizzard, duodenum, jejunum, ileum, cecum, and colon in broilers raised on two different diets in the presence and absence of AGPs. Samples were collected at three timepoints that represent distinct phases of poultry growth and dietary changes (days 10, 24, and 40). Previous poultry microbiome studies have largely focused on the ceca, due to its role in host health and high species richness [16, 40, 55–57]. Other intestinal sites have typically been neglected with only a limited number of studies utilizing material from feces [17, 58, 59] or the small intestine [25, 60, 61]. Furthermore, where studies have attempted more systematic comparisons, they have either focused on longitudinal sampling over one or two sites [17, 19] or sampling several intestinal sites at a single timepoint [25]. We also note that few studies have investigated the influence of AGPs in the context of wheat-based diets. Consequently, through sampling six sites over three timepoints, this study represents the first attempt to systematically perform a deep taxonomic and functional analysis of the dynamics of the chicken gut microbiome.

Focusing on community composition, our analyses revealed each site is associated with a discrete community, with the exception of the cecal and colon communities (Fig. 1D). Our findings are in broad agreement with previous studies with a dominance of lactobacilli in the upper GI, being replaced with obligate anaerobes, largely from the phylum, Firmicutes, in the lower GI. These latter taxa include *Lachnospiraceae* and other clostridia, which are associated with the processing of more complex carbohydrates and the production of short-chain fatty acids (SCFAs) that helps maintain gut health [45]. Interestingly, unlike several previous studies [25, 62, 63], members of the Bacteroidetes were not found to dominate the lower GI. However, while the emergence of Bacteroidetes is often associated with a more mature microbiome [16, 17], we note other studies also report the relatively low abundance of *Bacteroidetes* in older birds [40, 57]. Since both sets of reporting use similar diets, breeds, and methods of analysis, this suggests that the abundance of Bacteroidetes varies significantly between farms.

Results from our longitudinal analyses were more consistent with previous studies. For example, in ileal samples, we found an initial high abundance of *Lactobacillaceae* subsequently replaced by members of the Peptostreptococcaceae and Erysipelotrichaceae at later timepoints [19]. Similarly, we found the ceca to be first colonized by rapidly growing Enterobacteriaceae and subsequently replaced by slower growing taxa such as Clostridiales. For the upper GI, community dynamics were more limited, with little separation of the day 24 and 40 samples, from the day 10 samples (Supplemental Fig. 1). This highlights an important finding, namely that while microbial diversity in cecal datasets increased with age consistent with previous studies [7, 39, 56, 64], this was not the case for all sites and treatments (Supplemental Table 3). For example, under a corn diet supplemented with AGPs, the Shannon diversity of the jejunum appears to decrease with age. Thus, by focusing only on the cecal microbiome, treatments predicted to be beneficial to gut health may ignore potential adverse effects that occur at another GI site. Aside from site and time, diet also impacted community composition (Supplemental Fig. 1), likely reflecting differences in nutrient content [26, 27].

In contrast to other factors, we found that AGPs had only a modest impact on community composition. Of note however, we identified taxa, common to samples from both corn- and wheat-fed birds, which were impacted only under one of the diets. For example, in the day 40 cecal samples, 29 taxa exhibit altered abundance in both corn v wheat comparisons, as well as corn v corn + AGP comparisons, but are unaffected in wheat diets in the presence or absence of AGPs (Fig. 2A). Conversely, 19 taxa exhibit altered abundance in both corn v wheat comparisons, as well as wheat v wheat + AGP comparisons, but are unaffected in corn diets in the presence or absence of AGPs. This highlights the context of diet on the influence of AGPs and suggests that for many taxa, the effects of AGPs may be mediated through additional mechanisms, perhaps involving metabolism. For example, such effects may involve AGPs directly affecting an influential taxon (perhaps present in only one diet) that typically influences the growth or inhibition of other taxa (e.g., through cross-feeding or the production of inhibitory metabolites).

Supporting this idea is the finding that co-occurrence networks were reorganized in the presence of AGPs, with the disruption of interactions involving several influential taxa. This disruption is consistent with a previous study of cow rumen microbiomes, in which antibiotics decreased the number of interactions and connectivity of their co-occurrence networks [65]. These nuanced interactions between diet and AGP interaction may in part explain inconsistencies between studies that suggest either AGPs impact community structure (e.g., [18, 33, 66, 67]) or have only a minimal impact (e.g., [60, 64, 68]). Thus, in addition to site, studies attempting to identify additives that beneficially modulate the gut microbiome need to consider diet as a potential confounding factor.

Beyond community composition, it is important to investigate how AGPs might alter the function of the gut microbiome. To date, functional studies of the poultry gut microbiome have been limited to the application of metagenomics (whole microbiome shotgun DNA sequencing). Focusing on antimicrobial resistance genes, such studies reveal AGPs to have minimal impact on the abundance of such genes [69, 70]. However, in previous work, we used metatranscriptomics (whole microbiome RNASeq) to show that similar microbial communities in the mouse gut can exhibit dramatic differences in gene expression in different host genotypes [38]. Similarly, here we found that AGPs have a dramatic impact on the expression of microbial genes including many involved in metabolic pathways, cell wall biogenesis, and antimicrobial resistance.

Relative to cecal samples, we found that the jejunum samples contained more reads of host and diet origin, suggesting a lower density of bacteria. Encouragingly, taxonomic profiles were similar between the 16S RNA survey samples and the metatranscriptomic samples, albeit with certain taxa exhibiting different levels of activity, suggesting our pipelines are relatively robust in terms of taxonomic assignment. Pairwise comparisons revealed diet to have a greater impact on gene expression than AGPs, with changes in the expression of genes associated with several metabolic pathways, likely associated with differences in chemical composition in the two diets. Among the pathways altered by AGPs were those responsible for the production of energy and nucleotides. This is consistent with previous studies that have shown the following: (1) the murine gut microbiome alters metabolic pathway expression in the presence with antibiotics, with the effects driven by dietary components such as glucose and polysaccharides [49], and (2) bacteria respond to the stress of antibiotics through the diversion of ATP from core metabolic processes to drive the maintenance of ion gradients [48]. Beyond these general findings, we observed a complex landscape of metabolic pathway expression by different taxa in response to changes in diet and age. For example, many changes in glycolytic enzyme expression in the cecal microbiome of 24-day old birds are associated with *Lactobacillaceae* with a corn-diet and *Lachnospiraceae*, *Clostridiaceae*, and Enterobacteriaceae with a wheat-based diet. These findings raise an important consideration: to what extent are these changes in enzyme expression driven simply by changes in taxon abundance. We therefore compared fold-change differences in taxon abundance and global taxon expression with pathway-specific expression for individual taxa (see “Methods”). Focusing on three pathways discussed above (glycolysis, purine metabolism, and cell wall biogenesis), we found that pathway-specific expression for most taxa deviates by at least twofold from either changes in their abundance or global taxon gene expression (Supplemental Fig. 12). These findings suggest that changes in pathway expression are largely driven by differential regulation that is specific to those pathways.

While maps of metabolic pathways such as that presented in Fig. 6 provide a useful scaffold to reveal global taxonomic shifts in pathway expression, practical limitations in sequencing depth, together with loss of sequence conservation associated with poorly characterized taxa, limit our ability to reconstruct entire metabolic pathways for individual taxa. As a consequence, investigations focused on the metabolic contributions of individual taxa within a community remain challenging. Instead, complementary approaches such as community-based metabolic modeling offer alternative routes to systematically analyze metabolic interactions and cross-feeding relationships within a microbiome [50, 71].

Beyond the abrogation of the age-associated decrease in four metabolites associated with the urea cycle, we did not identify significant differences between AGP treatment and any of the metabolites measured in host serum. While it might be expected that host metabolite concentrations might correlate with the observed changes in expression of metabolic enzymes encoded by the microbiome, we note that our analysis only captured a limited number (139) of metabolites investigated. Furthermore, it has been shown that enzyme expression does not always translate to changes in metabolic flux [72], while the relationship between gut and serum metabolites also appears complex [73, 74].

In addition to metabolic interactions, which are reliant only on metabolites shared by enzymes and are therefore largely taxon agnostic, our ability to interpret metatranscriptomic data in the context of other bacterial systems is limited by the lack of appropriate resources. For example, the analysis of cell wall biogenesis genes presented in Fig. 8 relied on a high-quality network of interactions previously generated for *E. coli*. In addition to requiring the identification of homologs to *E. coli* genes, this analysis is unable to capture interactions representing taxon-specific innovations. To illustrate these issues, we constructed species-specific networks for the three most abundant taxa represented within the 24-day ceca samples from corn-fed birds (*Lactobacillus reuteri*, *Ruminococcaceae bacterium*, and *Lachnospiraceae bacterium*), using the STRING protein interaction database [75]. While we identified a core of conserved interactions involving homologs of genes involved in peptidoglycan biosynthesis, ftsZ, secA, and secE, each species lacked detectable homologs for many other genes (Supplemental Fig. 13). Furthermore, we note that many interactions are not conserved for each of the three taxa while also identifying additional interactions not captured by the *E. coli-*based network. This highlights the need for more taxon-agnostic scaffolds, providing generic representations of bacterial systems, while at the same time, capable of presenting taxon-specific innovations.

Previous studies have shown that DNA and RNA extraction methods can bias taxa recovered (e.g., [76]); we are nevertheless reassured that the extraction methods had a smaller impact on microbiome composition than biological factors such as sample collection site. For example, in Fig. 1, we find cecal and colon samples co-cluster, as do jejunum with duodenum samples. This is despite the DNA for these samples being extracted using different methods (TRIzol for ceca, DNeasy PowerSoil for colon and duodenum, RNA PowerSoil DNA elution kit for jejunum). An additional caveat to note in that although this study highlights several important microbial processes that respond to treatment with AGPs, in the absence of data showing absolute microbial abundance, we cannot rule out a major impact of AGPs being the lowering of abundance of bacteria in the GI microbiome. However, we do note that from our analysis of jejunum samples, a lowering of density of microbes might result in an increase in the abundance of host reads. No such increase was observed in the cecal samples from birds consuming AGPs.

In the search for treatments that may serve to replace AGPs, the findings from this study highlight several considerations that may be critical in developing efficacious alternatives. First, AGPs appear to redefine influential taxa in the microbiome that promote the presence and absence of other taxa. An obvious route to mimicking this effect would be to incorporate such “organizers” in probiotic formulations. Second, through altering microbial enzyme expression in pathways associated with nucleotide, amino acid, and carbohydrate and energy metabolism, AGPs may serve to redirect metabolic flux with the potential to regulate bacterial growth or produce metabolites that impact the host. As noted above, system-based approaches are required to further elucidate the downstream consequences of these alterations and identify alternative routes to effect such changes. Finally, the impact of AGPs is modulated by additional factors including age, diet, and GI site. Thus, solutions that are identified for, e.g., birds raised on one diet may not be effective for birds raised on an alternative diet. For example, if the upregulation of glycolysis in *Lactobacillaceae* provides a beneficial effect for birds fed a corn diet, the same effects may require upregulation of glycolysis in *Lachnospiraceae*, *Clostridiaceae*, and Enterobacteriaceae in birds fed a wheat diet.

## Conclusions

Previous studies of the impact of AGPs on the gut microbiome have largely focused on 16S rRNA surveys, revealing an inconsistent view of their impact on community composition. In this study, we highlight a more nuanced view of the impact of AGPs that is dependent on age of bird, diet, and intestinal site sampled. Applying metatranscriptomics, we show that the poultry GI microbiome responds to the presence of AGPs through changes in the expression of multiple biological systems, including the upregulation of pathways involved in cell wall biogenesis and antimicrobial resistance mechanisms, as well as more subtle changes in the expression of metabolic pathways, particularly those involved in energy, amino acid, and nucleotide metabolism. The wealth of novel data generated in this study represents a key advance towards understanding more causal relationships involving diet and the microbiome. Furthermore, we expect that some of the genes and pathways identified in this study will help drive future investigations that more closely examine the mechanistic effects that different diets impart on the microbiome. Of particular interest will be the application of system-based approaches such as community-based metabolic modeling tools, capable of dissecting these complex interrelationships.

## Methods

### Animal trials

Animal trials were carried out at the Poultry Research Center in Edmonton, Alberta, between November 2016 and January 2017. A total of sixty Ross 708 broilers were obtained from a commercial hatchery (Sofina Foods, Edmonton, AB). Post hatching, birds were allocated to 8 pens 21″ × 47″ × 17.5″ in size, and then, half of the birds were moved to new pens on day 11 so that 16 pens in total were occupied. The litter in each pen was wood shavings. Birds were fed a commercial starter diet until day 1 and then switched to a grower diet until day 24, and from day 25, the birds were fed a finisher diet (Supplemental Table 1). Corn-(typical of diets used in Ontario, Canada) and wheat-(typical of diets used in Alberta, Canada) based diets were formulated to meet or exceed all nutrient recommendations of the Ross 708 nutritional recommendations (Aviagen, Huntsville, Alabama, USA). Each pen was randomly assigned to a dietary treatment: corn-based diet, wheat-based diet, corn-based diet with 500 g/ton bacitracin methylene disalicylate (BMD; BMD110, Zoetis Canada, Kirkland QC) and 500 g/tonne monensin (Coban, Elanco Canada, Ltd., Guelph, ON) (Alpharma Canada Corporation, Mississauga, ON), and wheat-based diet with 500 g/tonne BMD and 500 g/ton monensin. AGPs were given to the birds in the AGP+ treatments during the entire trial. For the AGP-free diets (AGP−), 400 g/tonne of chemical coccidiostat (Nicarbazin, Nicarb 25%, Huvepharma, Sofia, Bulgaria) was added to the grower and finisher diets for wheat- and corn-based diets. Feed was prepared in-house with ingredients sourced from the University of Alberta feed mill.

Fifteen birds were allocated for each dietary regime, with five euthanized by cervical dislocation at three discrete timepoints: 10 days, 24 days, and 40 days of age. Intestinal wall and 5 ml washings were harvested from each bird’s gizzard, duodenum, jejunum, ileum, cecum, and colon (*N* = 5 for each tissue for each treatment/timepoint). Samples were flash frozen and sent for 16S rRNA survey sequencing and metatranscriptomics. A blood sample was drawn by brachial venipuncture prior to euthanasia (5 ml) from each bird, and serum was sent for targeted metabolomics at the Metabolomics Innovation Centre (University of Alberta).

### Nucleotide extraction

DNA was extracted from the gizzard, duodenum, ileum, and colon samples with Qiagen DNeasy PowerSoil kit, following the instructions of the manufacturer’s protocol. RNA was simultaneously extracted from jejunum and ceca samples for metatranscriptomics analyses. For jejunum samples, the Qiagen RNA PowerSoil kit was used for RNA extraction, and DNA extraction was performed simultaneously using the RNA PowerSoil DNA Elution Accessory kit to co-elute DNA from the extraction columns. The DNA was eluted in 50 uL of the elution buffer. For ceca samples, the PureLink TRIzol Plus Total Transcriptome protocol was followed for ceca RNA extraction, and total DNA was extracted simultaneously using the TRIzol reagent. Samples were thawed on ice, and 50–100 uL of sample was mixed with 1 mL of the TRIzol reagent. The TRIzol-sample mixture was vortexed for 30 s and incubated at room temperature for 5 min. RNA extraction was performed according to the PureLink RNA Mini Kit instructions, and DNA was isolated as described in the TRIzol Reagent protocol. The DNA pellets were resuspended in 300 uL of 8 mM NaOH. Some samples failed to yield DNA and/or RNA resulting in a final set of 336 DNA samples and 73 RNA samples (Supplemental Tables 2 and 5).

### 16S rDNA sequencing

The V4 hypervariable region of the 16S rRNA gene is amplified using a universal forward sequencing primer and a uniquely barcoded reverse sequencing primer to allow for multiplexing. Amplification reactions are performed using 12.5 uL of KAPA2G Robust HotStart ReadyMix (KAPA Biosystems), 1.5 uL of 10 uM forward and reverse primers, 8.5 uL of sterile water, and 2 uL of DNA. The V4 region was amplified by cycling the reaction at 95 °C for 3 min, 24× cycles of 95 °C for 15 s, 50 °C for 15 s, and 72 °C for 15 s, followed by a 5-min 72 °C extension. All amplification reactions were done in triplicate, checked on a 1% agarose Tris-borate-EDTA (TBE) gel, and then pooled in a single sample to reduce amplification bias. Pooled PCR product libraries were purified using 0.8× magnetic Ampure XP beads, selecting for the bacterial V4 amplified band. Purified libraries were quantified and sequenced by Illumina MiSeq, according to manufacturer instructions (Illumina, San Diego, CA). Sequencing was performed using the V2 (150 bp × 2) chemistry.

### Analysis of 16S rRNA sequence datasets

The UNOISE pipeline, available through USEARCH version 9.2 [77], was used for sequence analysis. The last base, typically error prone, was removed from all sequences. Sequences were assembled and quality trimmed using fastq_mergepairs and fastq_filter, with a fastq_maxee set at 1.0 and 0.5, respectively. Assembled sequences less than 233 bp were removed. Dereplication of sequences resulted in a set of unique sequences with corresponding abundances (number of reads matching each unique sequence). The unique set of sequences was then sorted, and singletons were removed using VSEARCH [78]. Resulting sequences were then denoised and chimeras filtered using the unoise2 command in USEARCH v. 9.2. The final set of sequences were then used for de novo OTU picking, using a 97% identity cutoff and using the otutab command. Taxonomy assignment of OTUs was executed by utax, available through USEARCH, using the Ribosomal Database Project (RDP) database version 16 [79], with a minimum confidence cutoff of 0.9. OTUs were aligned using PyNAST accessed through QIIME [80]. Sequences that did not align were removed from the dataset, and a phylogenetic tree of the filtered aligned sequence data was generated using FastTree [81]. Low abundance OTUs (< 0.005% relative abundance) were removed from subsequent analyses.

Downstream diversity analyses were performed using QIIME [80]. Alpha (within sample) diversity was calculated with the Shannon index. Samples were subsampled to 219 sequences per sample (the lowest number of sequences found in a single sample) to generate alpha rarefaction plots using the Shannon index to retain as many samples as possible. *T*-tests were used to assess whether alpha diversity was significantly different between groupings. Beta (between sample) diversity was measured using weighted UniFrac distances. Permutation multivariate analysis of variance (PERMANOVA) analyses with 9999 permutations were performed with the adonis function in the vegan R [82]. Permutational analysis of multivariate dispersions (PERMDISP) tests with 9999 permutations was performed in QIIME. All *p*-values were corrected for multiple comparisons using Benjamini and Hochberg’s false discovery rate method [83]. DESeq2 R package [47] was used to normalize raw OTU abundances with the “poscounts” method and to investigate whether any taxa were differentially abundant.

To examine main and interaction effects of AGPs and diet on the abundance of individual taxa (OTUs), we applied likelihood ratio tests on different negative binomial general linear models. In brief, likelihood ratio tests assess the significance of model terms by comparing the full model to a reduced model where those terms are missing. To assess the significance of diet main effects, a likelihood ratio test was performed for each taxon by comparing the performance of the model “taxon abundance~diet + AGPs” versus the reduced model “taxon abundance~AGPs.” To assess AGP main effects, a full model of “taxon abundance~diet + AGPs” was compared to the reduced model “taxon abundance~diet.” Finally, to access the significance of diet by AGPs interactions, a full model of “taxon abundance~diet + AGPs + diet × AGPs” was compared to the reduced model “taxon abundance~diet + AGPs.” False discovery rate (FDR) correction was performed on *p*-values across all taxa separately within each different likelihood ratio tests using the BH procedure (i.e., diet main effect, AGP main effect, and diet by AGP interaction), with an FDR cutoff of 5% (or 0.05) taken for statistical significance.

A correlation network was built from cecal content samples taken at day 40 using DGCA [44]. To reduce noise and complexity, the 1370 OTUs associated with the day 40 samples were grouped into 86 OTUs at the level of genus using phyloseq [84]. Next, we applied DESeq2 to normalize raw OTU abundances with the “poscounts” method [47]. The 86 OTUs were subsequently filtered to a set of 30 OTUs with DGCA’s recommended filtering function, filterGenes, using central tendency filtering (median method with a threshold set to the 30th percentile). DGCA was used to calculate Spearman correlation coefficients between OTUs within treatment groups; correlations with *p*-values less than 0.05 were considered significant. Additionally, DGCA calculated the difference of the z-transformed correlation values between treatment groups, yielding an adjusted *p*-value (Benjamini-Hochberg method) of the difference in z-scores. Adjusted *p*-values less than 0.05 were considered significant and were used to identify differential correlation between OTUs across different treatment groups. Visualization of the correlation network was performed using Cytoscape (v. 3.8.2) [85].

### Metatranscriptomic sequencing and analysis

RNA samples were depleted of human and bacterial ribosomal using RNA Ribo-Zero Gold rRNA Removal Kit (Epidemiology kit), followed by construction of libraries using the NEBNext® Ultra™ II RNA Library Prep kits. Sequencing was performed on a HiSeq 4000 platform at the Center for Applied Genomics (TCAG, Toronto, Canada) to generate ~30 million single end 150-bp reads per sample. Sequence reads were processed by our metatranscriptomic pipeline as described previously [36]. In brief, low quality and adaptor contaminants were trimmed using Trimmomatic v.0.36 [86] and VSEARCH v.2.4.4 [78], respectively. Duplicated reads were identified and removed by CD-HIT v.4.6.6 [87]. Next, host- and diet-associated reads were filtered using BWA [88], and BLAT [89] sequence similarity searches against the following: for host — the chicken genome and transcriptome (assembly Gallus_gallus-5.0 obtained from the National Centre for Biotechnology Information (NCBI; GCF_000002315.4 [90];) and ENSEMBL (release 90 [91];) respectively) and for diet — the corn, wheat, and soybean genomes (NCBI; GCF_000005005.2, GCA_900519105.1, and GCF_000004515.5). rRNA and tRNA reads were filtered using Infernal v.1.1.2 with the Rfam db (v13.0) [92]. The resulting reads of putative mRNA origin were then assembled into contigs using SPAdes v. 3.11.1 [93]. Contigs and unassembled reads were then annotated through a tiered set of similarity searches (BWA, BLAT, and DIAMOND [94]) against a database of sequenced microbial genomes (downloaded from NCBI October 2017; complete genomes and scaffolds), as well as the protein nonredundant database (downloaded from NCBI November 2017). After dereplication of sequence reads, transcripts with fewer than 5 reads were removed and expression levels normalized across samples with DEseq2 [46].

Transcripts were annotated with enzyme functions using our established pipeline [36] involving an ensemble method based on predictions obtained using DETECT [95], PRIAM [96], and Diamond v.0.9.10 [94] similarity searches against UniProt (downloaded may 2018 [97];). Protein-protein interaction data was obtained from a previously published study of functional interactions predicted for *E. coli* [53]. To map transcripts to this network, *E. coli* homologs were identified through DIAMOND sequence similarity searches filtering for matches that were either greater than 100 bp in length with bit score greater than 60 or had sequence identity greater than 85% with a percentage of overlap greater than 65.

To examine main and interaction effects of AGPs and diet on gene expression, we applied the same likelihood ratio test framework as described above for taxa abundance. For these analyses, genes were filtered for those found with at least 5 counts in at least 4 samples. As before false discovery rate (FDR), correction was performed on *p*-values across all genes separately within each different likelihood ratio test using the BH procedure (i.e., diet main effect, AGP main effect, and diet by AGP interaction), with an FDR cutoff of 5% (or 0.05) taken for statistical significance.

### Metabolomics processing

From the collected serum samples, metabolomics profiling was performed for 139 metabolites by The Metabolomics Innovation Centre (TMIC; University of Alberta, Edmonton, AB) using their TMIC Prime Metabolomics Profiling Assay service. MetaboAnalyst [98] was used for data analysis, AGP-treated samples were normalized to sample D40-18, and control samples were normalized to sample D10-13; the normalization parameters were “SamplePQN” and “AutoNorm.” Spearman rank was used to correlate metabolite profiles to the pattern “1-2-3” to identify metabolites correlated with age using the “Match.Pattern” function. PCA was conducted using standard parameters with the “PCA.Anal” function. To investigate diet differences, antibiotic differences, and diet by antibiotic interactions in the metabolomics data, a permutation-based factorial ANOVA was used. Analyses were run separately for each metabolite. The BH procedure was used to correct for FDR within main effect and interaction terms across all metabolites, and FDR < = 0.05 was taken as significant. Permutation ANOVA tests were implemented using the analysis function aovp in the lmPerm R package (setting Ca = 1/1,000,000, maxIter = 1,000,000).

### Additional statistical analyses of metatranscriptomics data

#### Principle component analyses

To reveal the correlation of the overall expression distributions relating to transcripts across samples, we applied principal component analysis (PCA) using the prcomp function from R [82]. PERMANOVA [99] tests were applied to analyze treatment separation, implemented through the f_npManov function of the MATLAB (R2015a, The MathWorks Inc., Natick, MA, USA) toolbox Fathom [100], using 100,000 replicate label permutations and adjusting *p*-values with the Benjamini-Hochberg procedure [83]. The cutoff of the adjusted *p*-value was set as 0.05.

#### Differential expression analysis of transcripts and enzymes

Differential expression analysis of mapped transcripts for pairwise comparisons was performed using DESeq2 [47]. Significantly differentially expressed transcripts were defined as those assigned *q*-values < 0.05 (using the Benjamini-Hochberg procedure to correct *p*-values), together with log2 fold change in expression between samples greater than 1. Enzymes were defined as significantly differentially expressed if at least one significantly differentially expressed transcript was mapped to that enzyme.

#### Gene set enrichment analysis of metabolic pathways

To test if KEGG-defined pathways [101] were enriched with significantly differentially expressed enzymes, gene set enrichment analyses (GSEA) were performed using a hypergeometric test with a minimum of two genes per gene set. In these analyses, to ensure consistency across sample comparison, we examined enrichment relative to the total pool of all transcripts identified across all ceca or jejunum samples. We used a false discovery rate (FDR) adjustment with the Benjamini-Hochberg procedure to correct *p*-values. Hypergeometric tests were performed using the hygecdf and mafdr functions from MATLAB with a FDR cutoff of 0.05.

#### Contribution of taxon abundance and global gene expression on changes in pathway-specific expression

To examine the influence of taxon abundance and global gene expression for a taxon on differences observed in the expression of three selected pathways (glycolysis, purine metabolism, and cell wall biogenesis), we first calculate taxon-specific changes of gene expression for each of the three pathways (as measured by the mean log2 fold change of all genes in the pathway for that taxon). These calculations were performed for each of the four treatment comparisons: corn + AGPs v corn, wheat + AGPs v wheat, corn v wheat, and corn + AGPs v wheat + AGPs. We then calculated the absolute difference of these pathway-specific values to the following: (1) the relative change in abundance of that taxon (as measured by the log2 fold change associated with that taxon according to its 16S rDNA-based relative abundance) and (2) the total change in RNA for that taxon (as measured by the log2 fold change in total taxon RNA, calculated by summing RPKM values for all genes for that taxon). Values close to zero indicate that the shift in pathway gene expression was driven by a general shift in either taxon abundance (16S) and/or global taxon expression. Single sample Wilcoxon tests were then applied to determine if the median difference in fold change for all taxa associated with that pathway was greater than 2. For these analyses, OTUs are grouped into genera; thus, each taxon represents an individual genus.

## Supplementary Information

**Additional file 1: Supplemental Figure 1**. PCoA plots of weighted UniFrac distances for samples associated with each gastrointestinal site. Samples are coloured according to time of sampling. Sample shape represents diet.

**Additional file 2: Supplemental Figure 2**. Heatmap showing normalized abundance of taxa for taxa exhibiting significant differential abundance in at least one of the comparisons shown on the right. Each column in the heatmap represents a single sample, grouped by treatment. Each row indicates an individual taxon. Columns on the right indicate significant differences in abundance across each of eight pair-wise comparisons. Taxa are ordered on the basis of magnitude of fold change across the various pairwise comparisons.

**Additional file 3: Supplemental Figure 3**. Pie-chart heatmap of metabolic pathways enriched with significantly differentially expressed enzymes through comparisons between diets. There are 35 KEGG metabolic pathways enriched with significantly differentially expressed enzymes which were annotated from either ceca or jejunum samples based on comparisons between diets. Each node in the heatmap is a pie chart showing proportion of enzymes that were significantly up-regulated (orange), significantly down-regulated (blue), and not significantly different (white) with the wheat diet. In addition, the size of each node indicates the number of expressed enzymes in each KEGG pathway.

**Additional file 4: Supplemental Figure 4**. Principal component analysis based on annotated metabolic enzymes expression in ceca samples. Each node represents an individual ceca sample, with colours and shapes indicating specific treatment types (see inset key).

**Additional file 5: Supplemental Figure 5**. Expression heatmap of metabolic pathways enriched in significantly differentially expressed enzymes. There are 35 KEGG metabolic pathways enriched in significantly differentially expressed enzymes associated with samples from either ceca or jejunum. Changes of the average expression value (log2) of each gene associated with an enzyme in that pathway are indicated by red-green or blue-yellow gradients for ceca and jejunum samples, respectively.

**Additional file 6: Supplemental Figure 6**. Taxonomic contributions to expressed enzymes in metabolic pathways across different AGP treatments and diets with Day40 ceca samples. Glycolysis/Gluconeogenesis, Pentose Phosphate and Tricarboxylic acid (TCA) cycle pathways are shown here integrated with data generated from cecal samples collected at day 40. Each pie chart represents the taxonomic distributions of an enzyme (see key for color code). The size of pie charts indicates the average expression value (with log2 transform) of genes encoding that enzyme. Pie charts with red arrows refer to enzymes that are significantly up-regulated relative to the paired (+/- AGPs) sample. The abbreviations used here can be found in Supplemental Table 11.

**Additional file 7: Supplemental Figure 7**. Taxonomic contributions to expressed enzymes in Purine metabolic pathway across different AGP treatments and diets with Day24 ceca samples. Shown here are the *De novo* biosynthesis and salvage pathways for purine, integrated with data generated from ceca collected at day 24. Each pie chart represents the taxonomic distributions of an enzyme (see key for color code). The size of pie charts indicates the average expression value (with log2 transform) of genes encoding that enzyme. Pie charts with red arrows refer to enzymes that are significantly up-regulated relative to the paired (+/- AGPs) sample. The abbreviations used here can be found in Supplemental Table 11.

**Additional file 8: Supplemental Figure 8**. Taxonomic contributions to expressed enzymes in Purine metabolic pathway across different AGP treatments and diets with Day40 ceca samples. Shown here are the *De novo* biosynthesis and salvage pathways for purine, integrated with data generated from ceca collected at day 40. Each pie chart represents the taxonomic distributions of an enzyme (see key for color code). The size of pie charts indicates the average expression value (with log2 transform) of genes encoding that enzyme. Pie charts with red arrows refer to enzymes that are significantly up-regulated relative to the paired (+/- AGPs) sample. The abbreviations used here can be found in Supplemental Table 11.

**Additional file 9: Supplemental Figure 9**. AGPs disrupt age related changes in metabolite profiles from chicken serum. PCA of metabolomic profiles generated from either AGP+ or AGP- samples. Samples are coloured and grouped based on age of bird from which the sample was taken.

**Additional file 10: Supplemental Figure 10**. Taxonomic contributions to gene expression profiles for proteins involved in cell wall biogenesis for ceca samples collected at day 40. Each node in the network indicates groups of orthologs corresponding to a specific *E. coli* gene (as indicated) involved in cell wall biogenesis. Links between nodes indicate a functional interaction as previously defined [53]. Size of the node indicates the relative expression of genes associated with each set of orthologs, with sector colours indicating the taxonomic contribution to gene expression (see key for color code). Red arrows indicate sets of orthologs that are significantly up-regulated in comparisons involving the presence/absence of AGPs.

**Additional file 11: Supplemental Figure 11**. Taxonomic contributions to gene expression profiles for proteins involved in cell wall biogenesis for jejunum samples collected at day 40. Each node in the network indicates groups of orthologs corresponding to a specific *E. coli* gene (as indicated) involved in cell wall biogenesis. Links between nodes indicate a functional interaction as previously defined [53]. Size of the node indicates the relative expression of genes associated with each set of orthologs, with sector colours indicating the taxonomic contribution to gene expression (see key for color code). Red arrows indicate sets of orthologs that are significantly up-regulated in comparisons involving the presence/absence of AGPs.

**Additional file 12: Supplemental Figure 12**. Comparison of changes in pathway-specific gene expression, with changes in taxon abundance and global gene expression for three select pathways. For each pathway (glycolysis, purine metabolism and cell wall biogenesis) we calculate the shift in expression across the four conditions tested (i.e. corn + AGPs v corn; wheat + AGPs v wheat; corn v wheat; and corn + AGPs v wheat + AGPs). The absolute deviation of these changes in pathway-specific expression were then calculated relative to: 1) the relative change in abundance of that taxon (as measured by the log2 fold-change associated with that taxon according to 16S rDNA-based relative abundance); and 2) the total change in RNA for that taxon (as measured by the log2 fold-change in total taxon RNA, calculated by summing RPKM values for all genes for that taxon). Here OTUs are grouped into genera. In the box-plots, taxa with values close to zero, indicate that the shift in pathway gene expression was driven by a general shift in either taxon abundance (16S) and/or global taxon expression. Of the 48 comparisons presented, 42 show that at least 60% of taxa with pathway expression, deviate by greater than a log2 fold change from changes in relative abundance (as measured by 16S rDNA) or global taxon expression (as measured by total taxon RPKM). Note, due to lack of phylogenetic resolution associated with the 16S rDNA datasets, these comparisons feature fewer taxa than the comparisons involving taxon-specific global gene-expression. Single sample Wilcoxon tests further reveal that for 43 of the datasets, pathway-specific gene-expression significantly deviates from either taxon-abundance or global gene expression (* *p*<0.05; ** *p*< 0.01; *** *p*<0.001). The lower panels show the 20 genera exhibiting the greatest divergence in pathway expression relative to global gene expression. For the Corn v Corn+AGP comparison for the glycolysis pathway, we identified ‘Other Bacteria’ and various Proteobacterial groups as possessing the greatest deviation between pathway-specific and global fold change expression. For the Wheat v Wheat+AGP comparison. among the taxa with the greatest deviation between pathway-specific and global fold change expression, were taxa classified as ‘Other Bacteria’, ‘Peptostreptococcaceae’ and ‘Clostridiaceae’.

**Additional file 13: Supplemental Figure 13**. Taxon-specific expression profiles for genes encoding proteins involved in cell wall biogenesis for ceca samples collected at day 24. Each network represents proteins and their interactions associated with the three select taxa. Nodes represent an ortholog of an *E. coli* protein previously predicted to be involved in cell wall biogenesis [53]. Size of the node indicates the relative expression of genes associated with the ortholog for that taxon. Links between nodes indicate a functional interaction as defined by the STRING protein interaction database [75], with those in green also found in the previously described *E. coli* network, and those in orange, not found in the previously described *E. coli* network.

**Additional file 14: Supplemental Table 1**. Ingredient and calculated nutrient composition of experimental broiler chicken diets.

**Additional file 15: Supplemental Table 2**. Details of Samples and 16S Survey Sequencing Statistics.

**Additional file 16: Supplemental Table 3**. Shannon diversity indices of samples classified according to age, site, and treatment. Averages of each grouping are shown with standard deviation in brackets.

**Additional file 17: Supplemental Table 4**. Abundance of Taxa in Cecal Samples Exhibiting Significant Changes in Abundance Across Different Treatments.

**Additional file 18: Supplemental Table 5**. Details of Metatranscriptome Sequencing Statistics.

**Additional file 19: Supplemental Table 6**. List of bacterial transcripts and their relative expression (RPKM) across all samples.

**Additional file 20: Supplemental Table 7**. Main and Interaction Effects of Diet and AGPs on Gene Expression for Day24 and Day40 Cecal Samples.

**Additional file 21: Supplemental Table 8**. Transcripts expressed in the ceca exhibiting significant differential expression between AGPs and diets.

**Additional file 22: Supplemental Table 9**. Metabolic pathways enriched in transcripts expressed in the ceca exhibiting significant differential expression between AGPs and diets.

**Additional file 23: Supplemental Table 10**. Enzymes detected in 73 metatranscriptomic datasets.

**Additional file 24: Supplemental Table 11**. List of Abbreviations of Compounds and Enzymes.

**Additional file 25: Supplemental Table 12**. Metabolite profiles of serum samples collected from all 60 birds used in the study. Supplemental Table 12 Metabolite profiles of serum samples collected from all 60 birds used in the study.

**Additional file 26: Supplemental Table 13**. Expression of transcripts (RPKM) associated with antimicrobial resistance mechanisms across cecal samples.

**Additional file 27: Supplemental Table 14**. Expression of transcripts (RPKM) associated with antimicrobial resistance mechanisms across jejunum samples.

## Data Availability

Sequence data is available at the NCBI Sequence Read Archive (https://www.ncbi.nlm.nih.gov/sra/) with the BioProject identifier: PRJNA614900.
