# Supplementary figures and images for: Systematic profiling of the chicken gut microbiome reveals dietary supplementation with antibiotics alters expression of multiple microbial pathways with minimal impact on community structure
Source: Microbiome. 2022 Aug 15;10:127. doi: 10.1186/s40168-022-01319-7 (PMC9377095; doi:10.1186/s40168-022-01319-7)

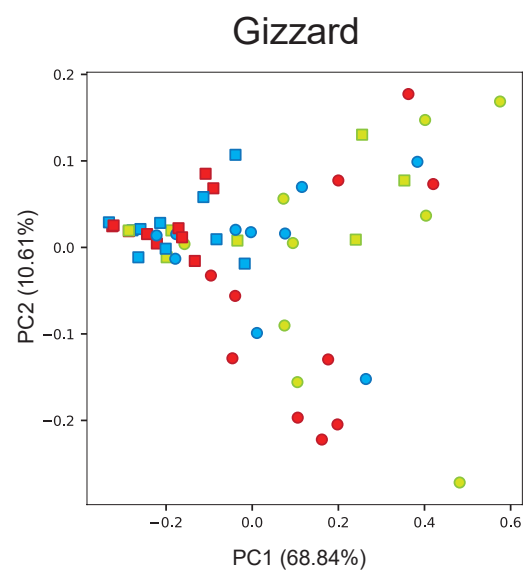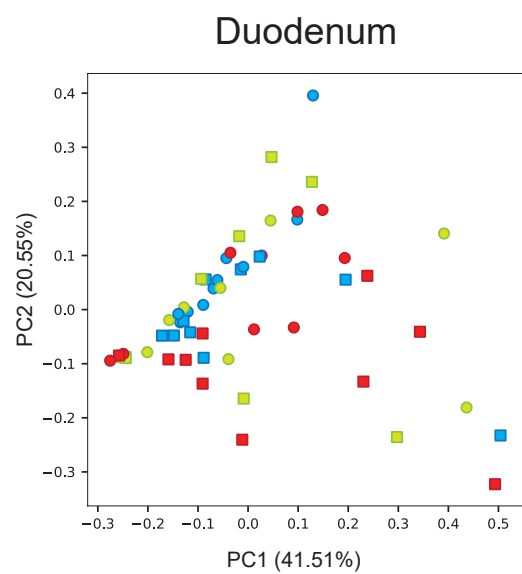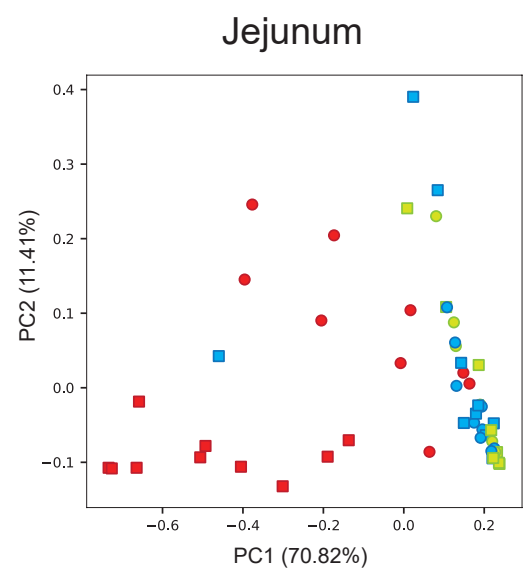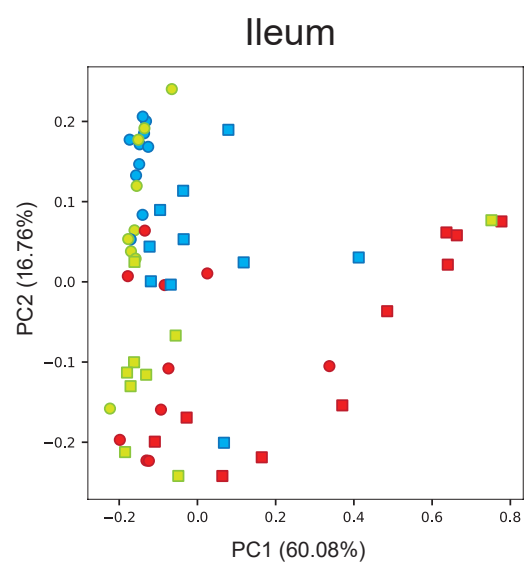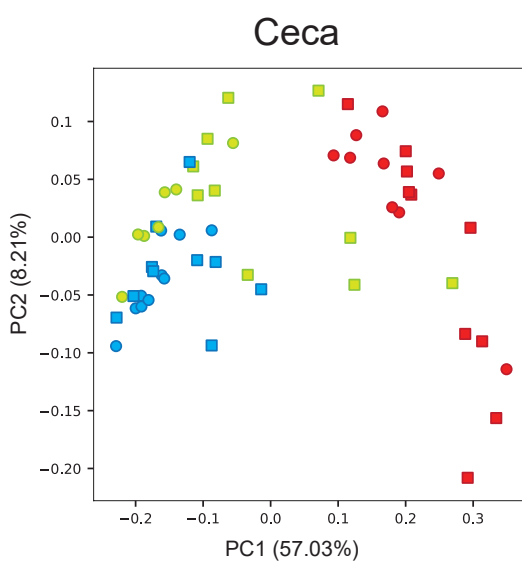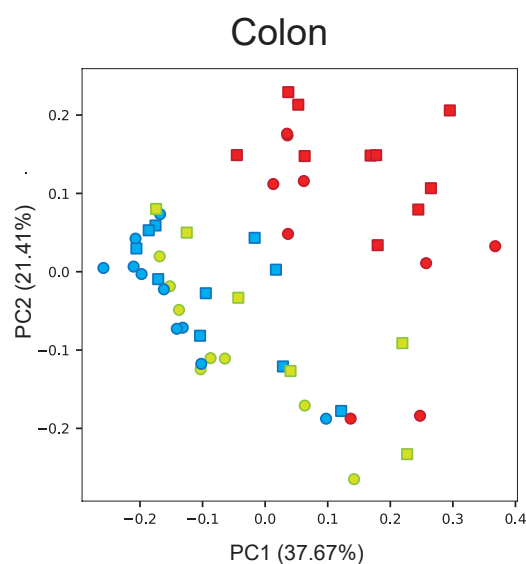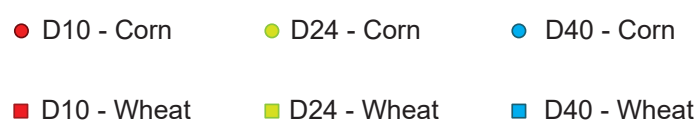

Supplement: Supplementary file 2 — Additional file 1: Supplemental Figure 1. PCoA plots of weighted UniFrac distances for samples associated with each gastrointestinal site. Samples are coloured according to time of sampling. Sample shape represents diet. [file 40168_2022_1319_MOESM1_ESM.pdf]

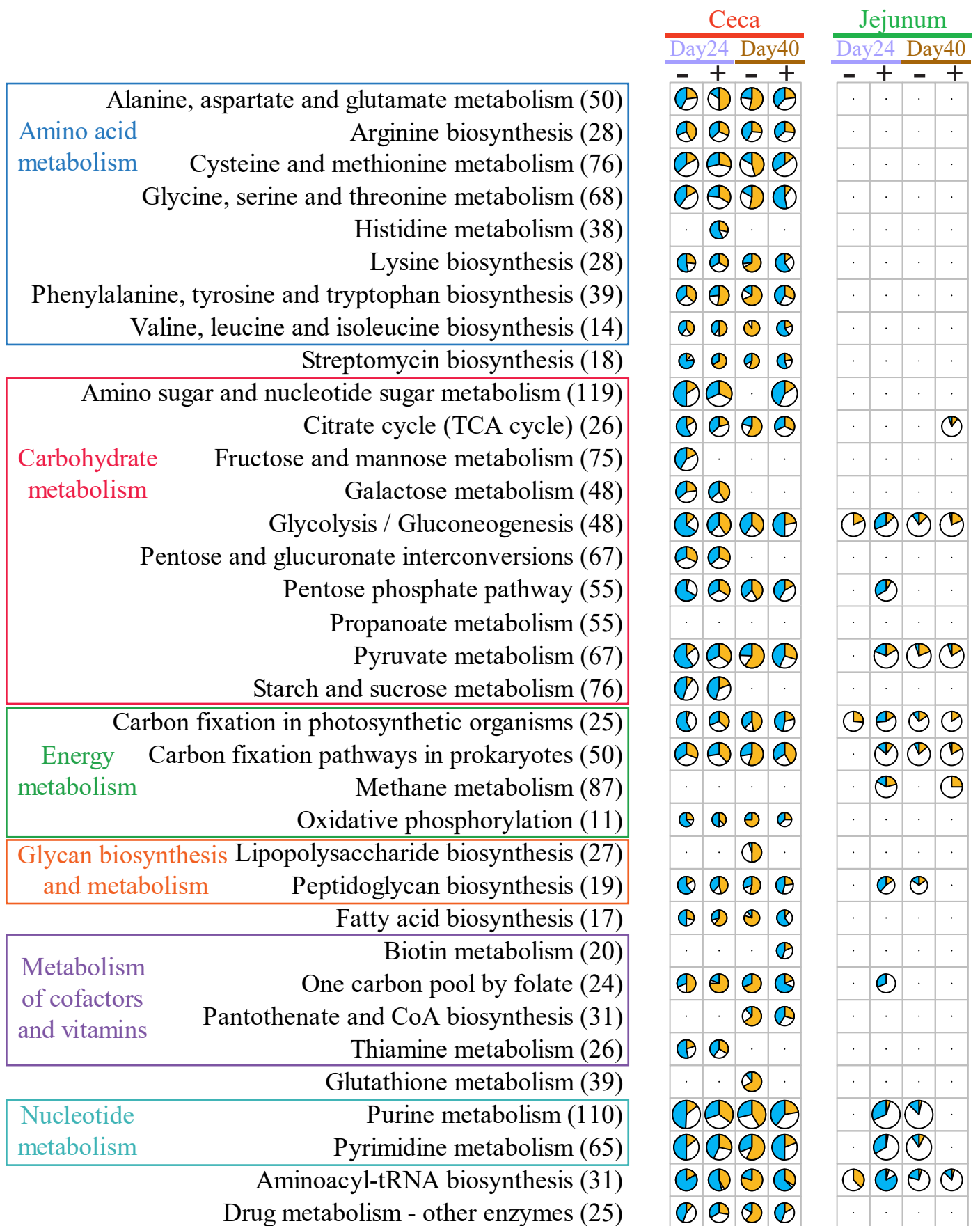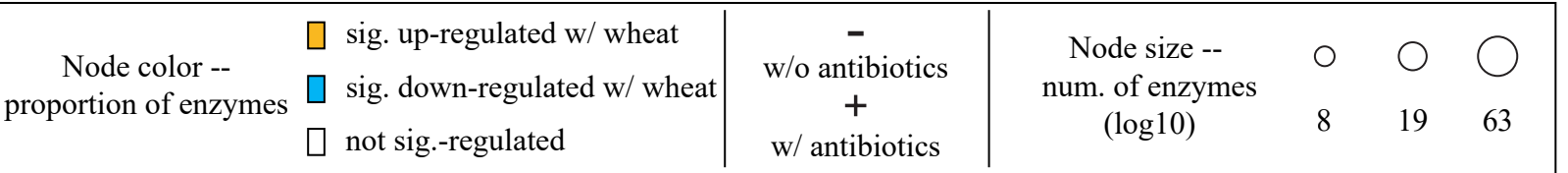

Supplement: Supplementary file 4 — Additional file 3: Supplemental Figure 3. Pie-chart heatmap of metabolic pathways enriched with significantly differentially expressed enzymes through comparisons between diets. There are 35 KEGG metabolic pathways enriched with significantly differentially expressed enzymes which were annotated from either ceca or jejunum samples based on comparisons between diets. Each node in the heatmap is a pie chart showing proportion of enzymes that were significantly up-regulated (orange), significantly down-regulated (blue), and not significantly different (white) with the wheat diet. In addition, the size of each node indicates the number of expressed enzymes in each KEGG pathway. [file 40168_2022_1319_MOESM3_ESM.pdf]

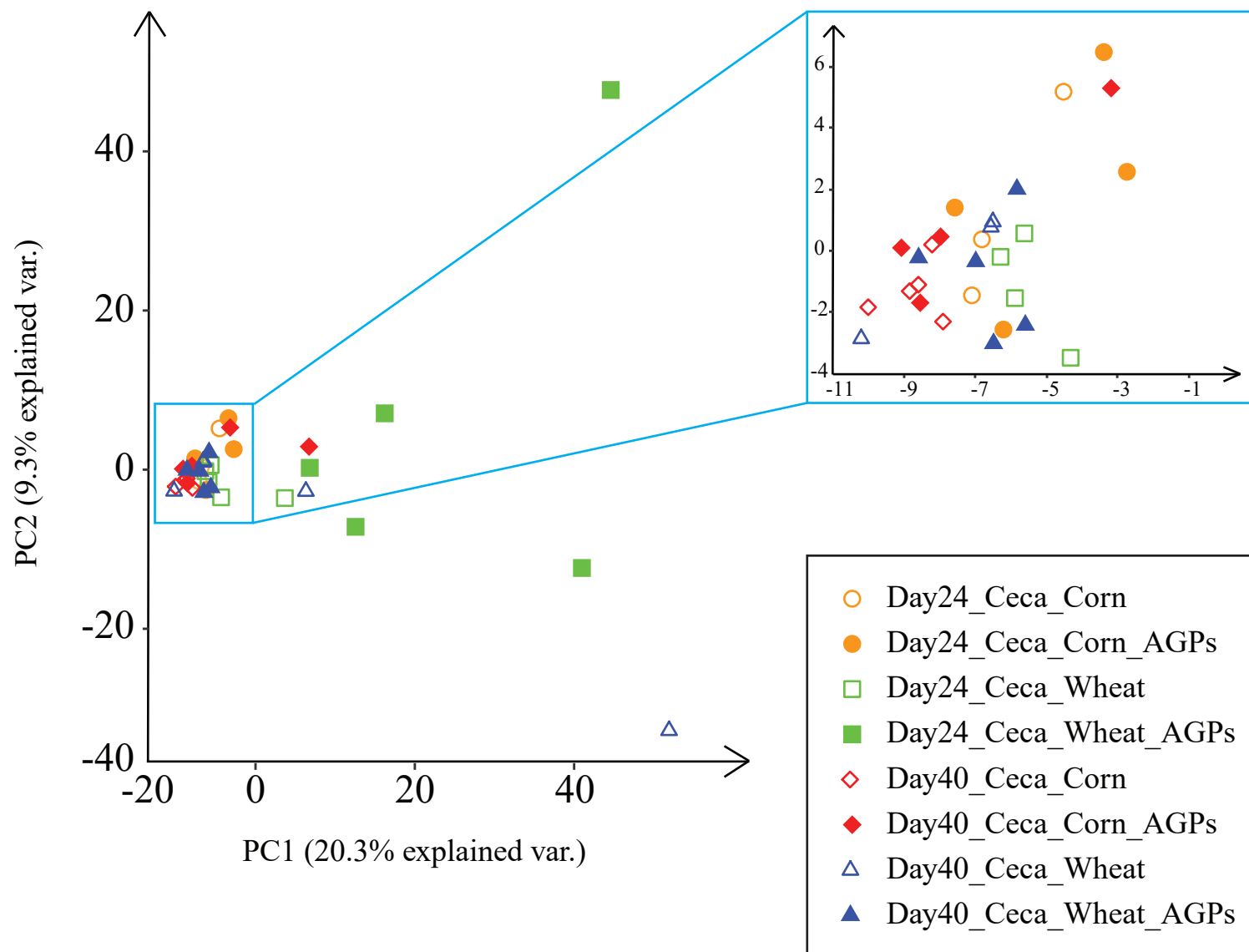

Supplement: Supplementary file 5 — Additional file 4: Supplemental Figure 4. Principal component analysis based on annotated metabolic enzymes expression in ceca samples. Each node represents an individual ceca sample, with colours and shapes indicating specific treatment types (see inset key). [file 40168_2022_1319_MOESM4_ESM.pdf]

## Day24 Corn

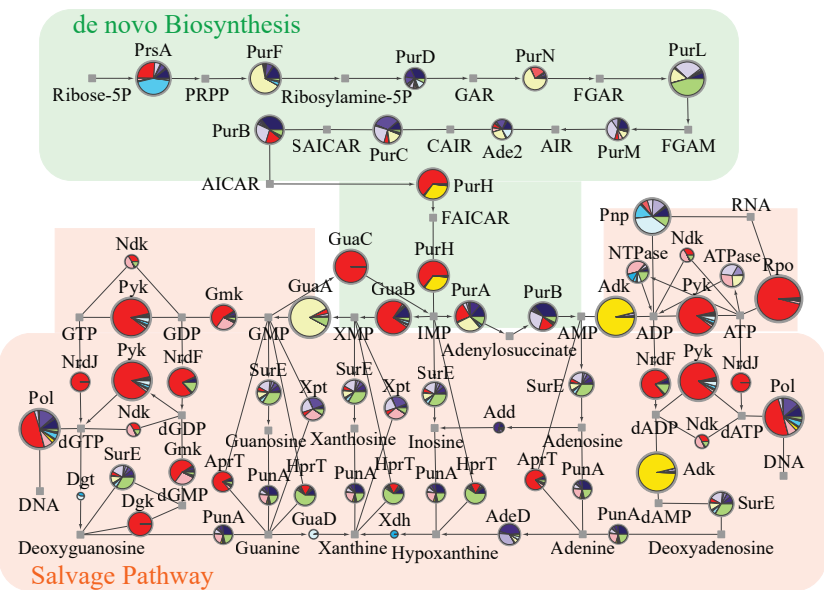

## Day24 Wheat

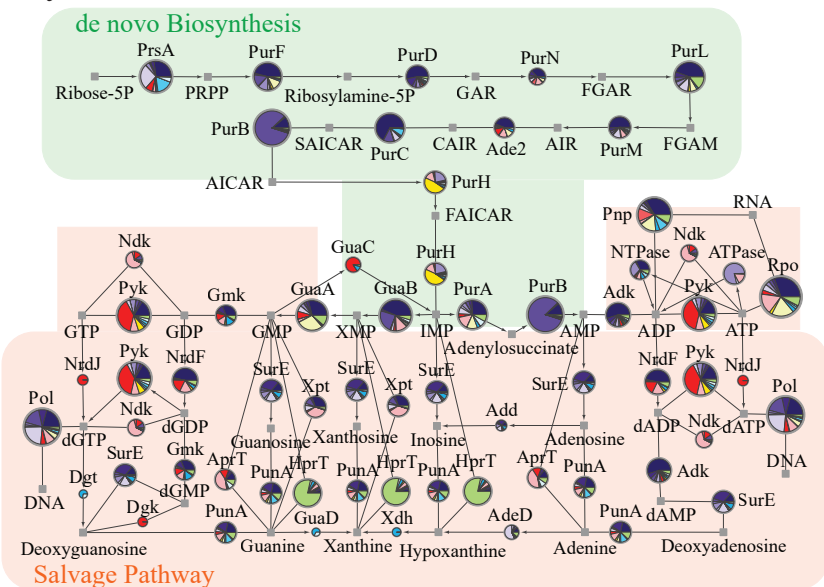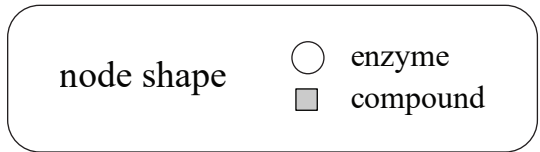

## Day24 Corn + AGPs

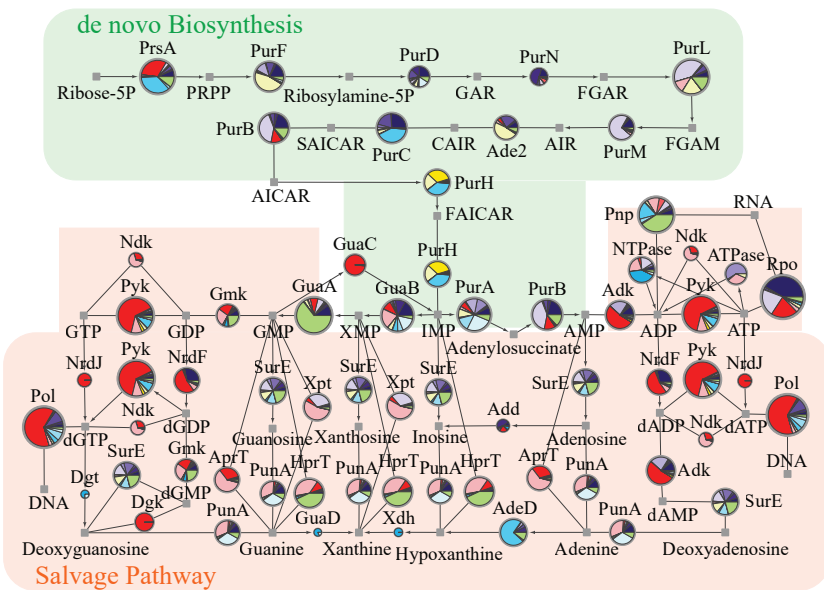

## Day24 Wheat + AGPs

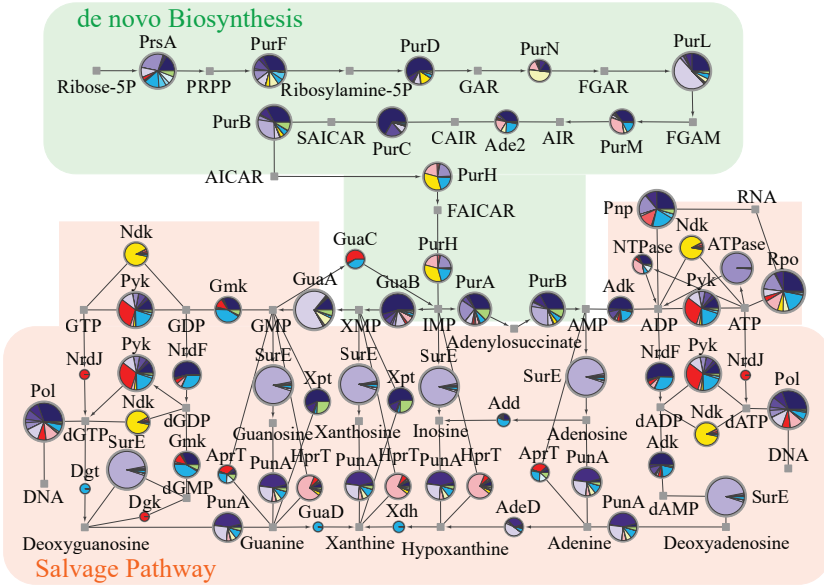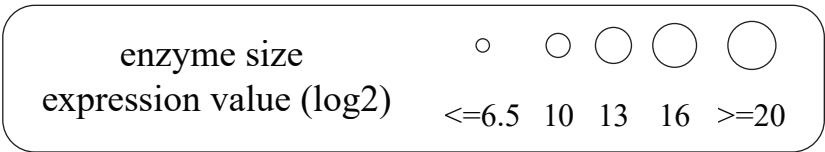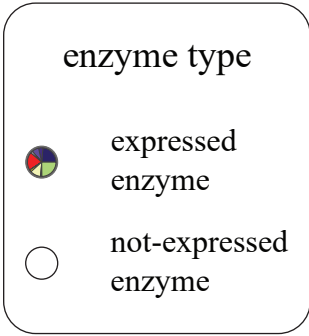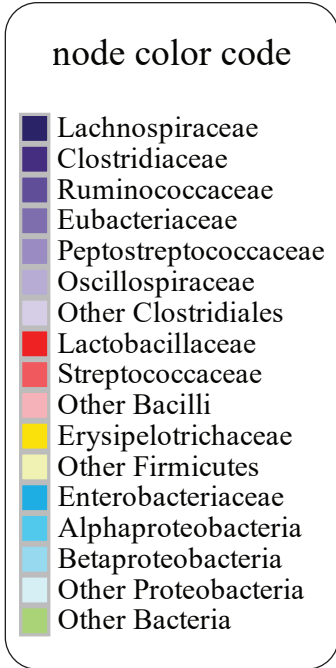

Supplement: Supplementary file 8 — Additional file 7: Supplemental Figure 7. Taxonomic contributions to expressed enzymes in Purine metabolic pathway across different AGP treatments and diets with Day24 ceca samples. Shown here are the De novo biosynthesis and salvage pathways for purine, integrated with data generated from ceca collected at day 24. Each pie chart represents the taxonomic distributions of an enzyme (see key for color code). The size of pie charts indicates the average expression value (with log2 transform) of genes encoding that enzyme. Pie charts with red arrows refer to enzymes that are significantly up-regulated relative to the paired (+/- AGPs) sample. The abbreviations used here can be found in Supplemental Table 11. [file 40168_2022_1319_MOESM7_ESM.pdf]

Day40 Corn

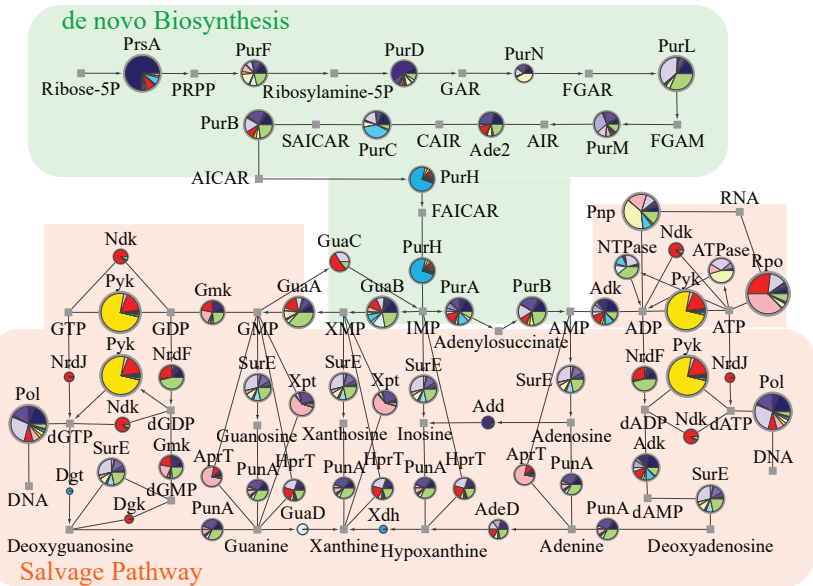

Day40 Corn + AGPs

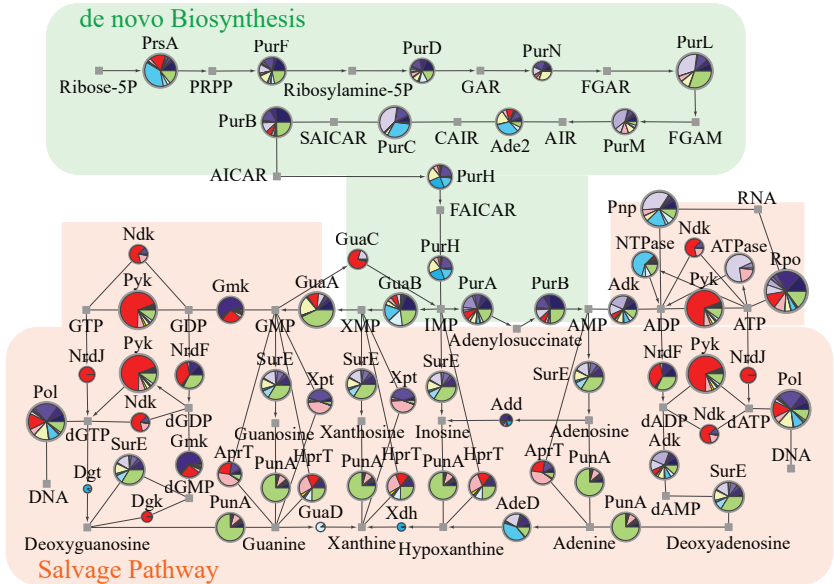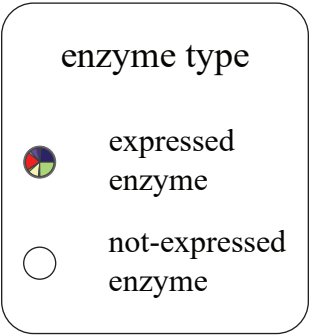

Day40 Wheat

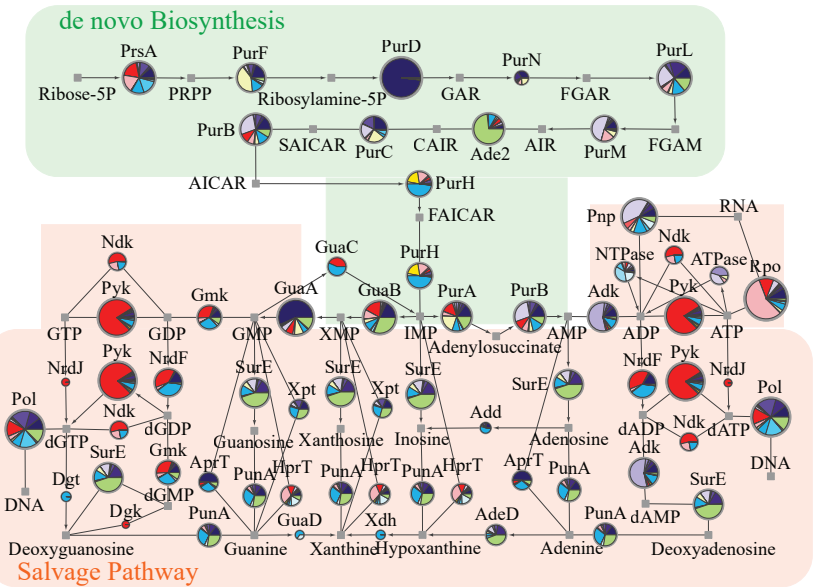

Day40 Wheat + AGPs

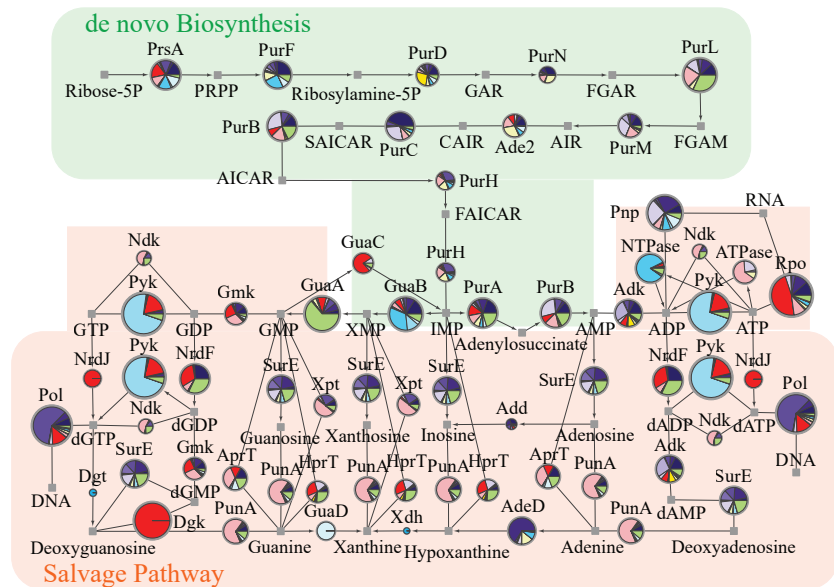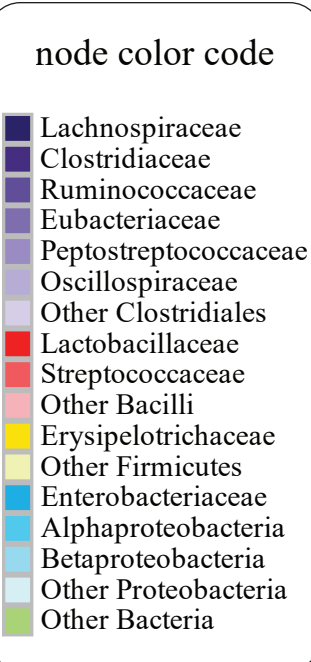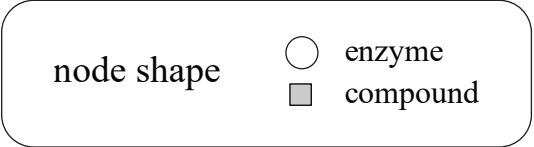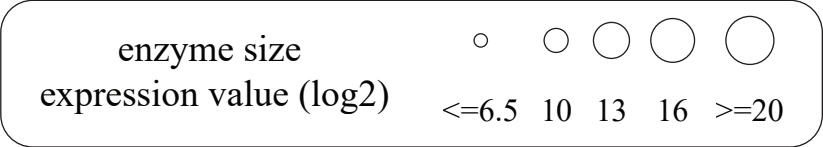

Supplement: Supplementary file 9 — Additional file 8: Supplemental Figure 8. Taxonomic contributions to expressed enzymes in Purine metabolic pathway across different AGP treatments and diets with Day40 ceca samples. Shown here are the De novo biosynthesis and salvage pathways for purine, integrated with data generated from ceca collected at day 40. Each pie chart represents the taxonomic distributions of an enzyme (see key for color code). The size of pie charts indicates the average expression value (with log2 transform) of genes encoding that enzyme. Pie charts with red arrows refer to enzymes that are significantly up-regulated relative to the paired (+/- AGPs) sample. The abbreviations used here can be found in Supplemental Table 11. [file 40168_2022_1319_MOESM8_ESM.pdf]

Control

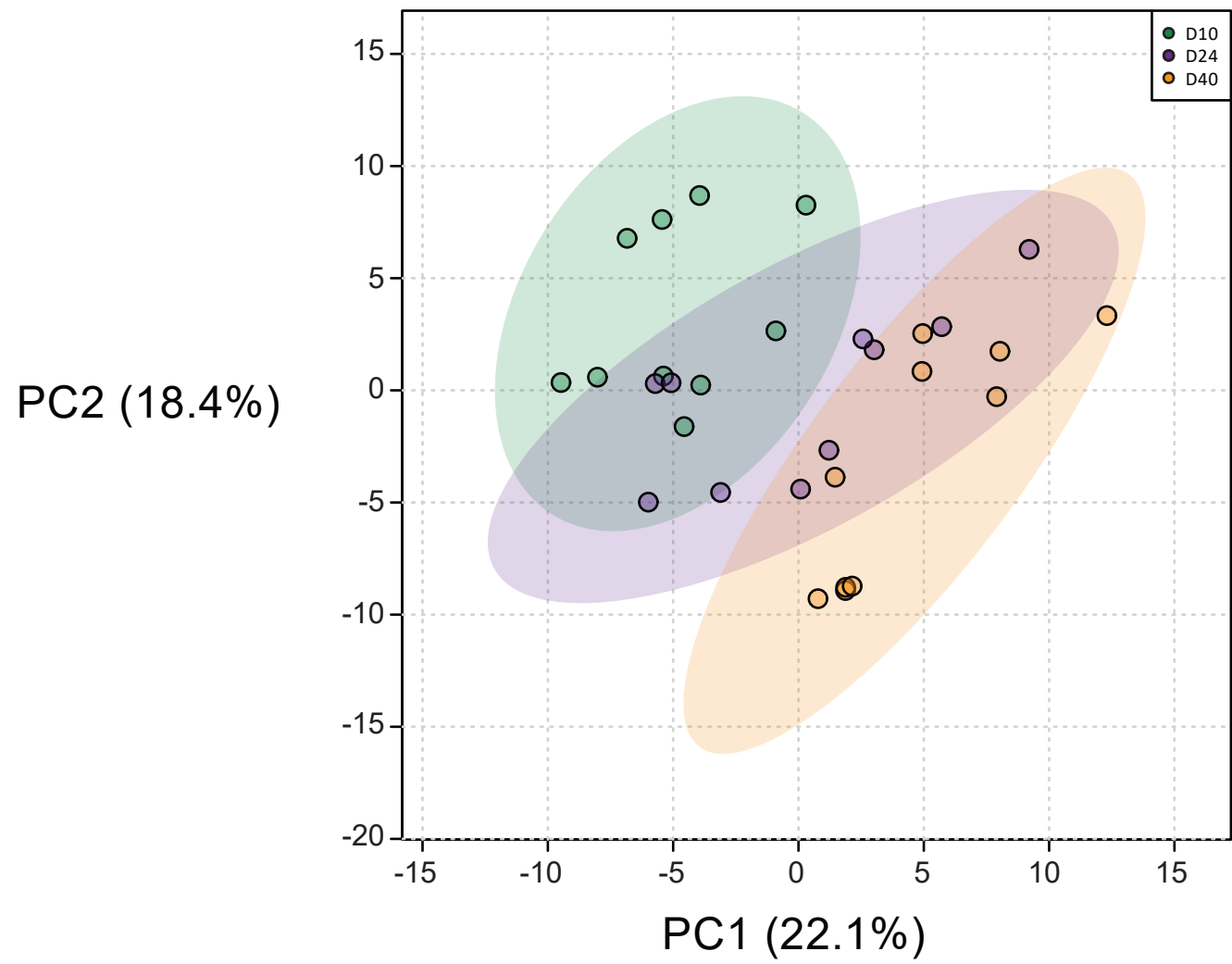

Antibiotics

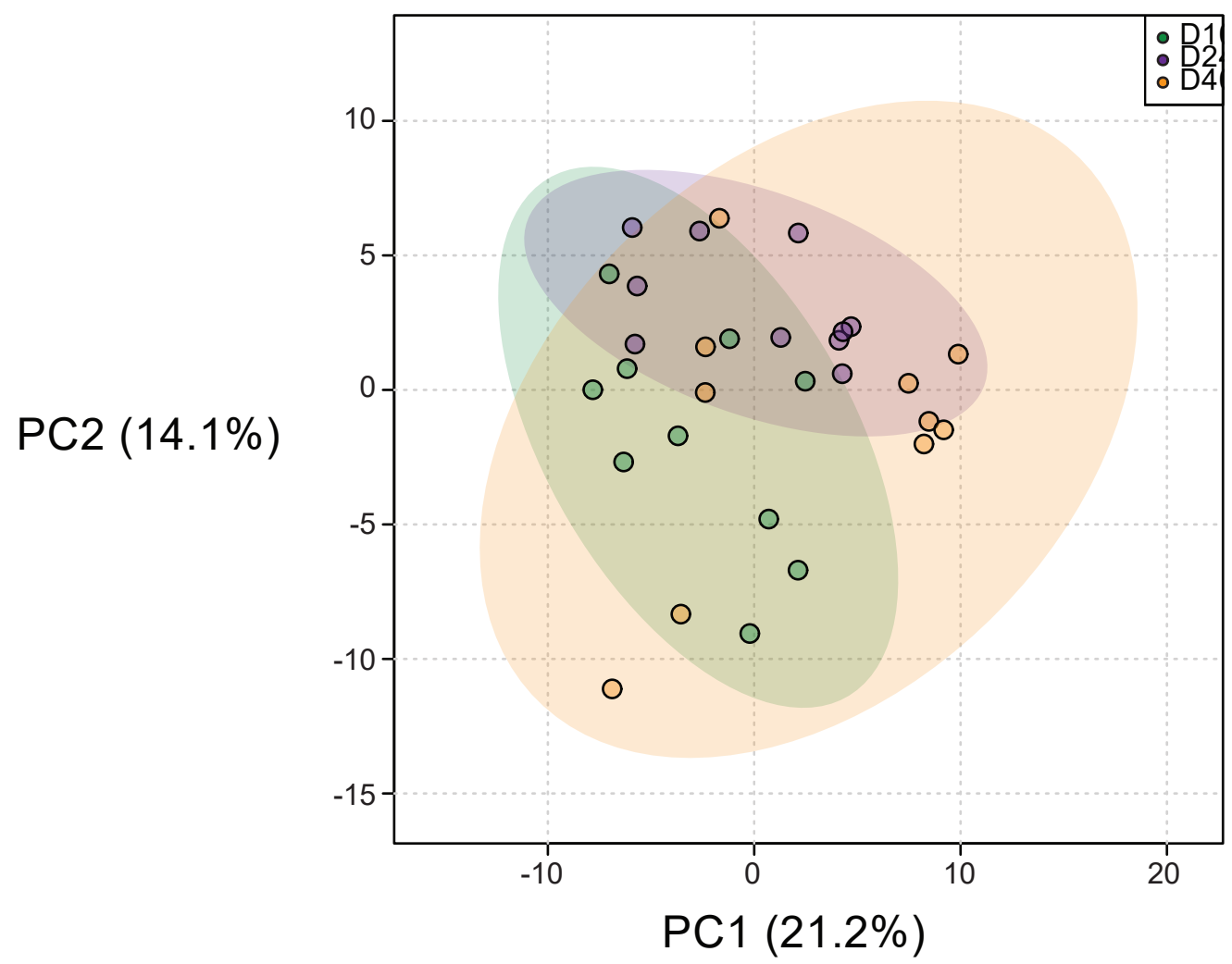

Supplement: Supplementary file 10 — Additional file 9: Supplemental Figure 9. AGPs disrupt age related changes in metabolite profiles from chicken serum. PCA of metabolomic profiles generated from either AGP+ or AGP- samples. Samples are coloured and grouped based on age of bird from which the sample was taken. [file 40168_2022_1319_MOESM9_ESM.pdf]

(A) Day 40 Ceca / Wheat

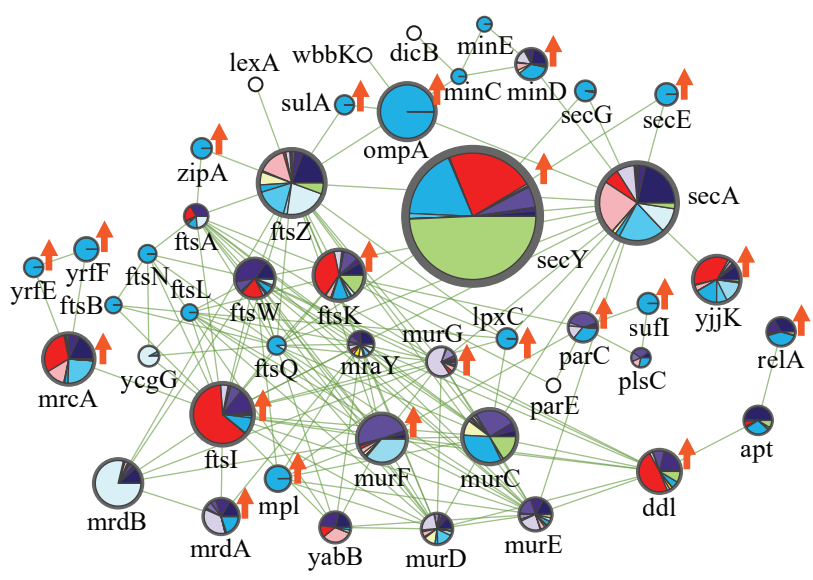

(B) Day 40 Ceca / Wheat / AGPs

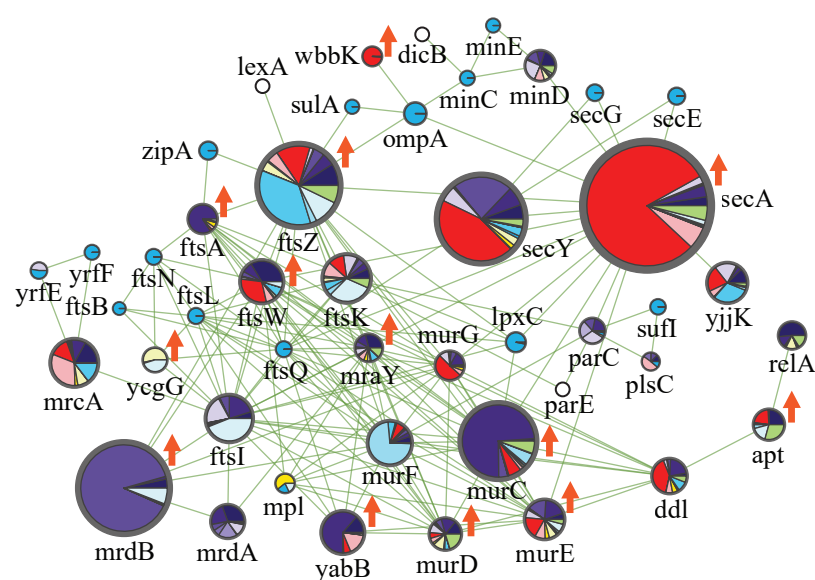

(C) Day 40 Ceca / Corn

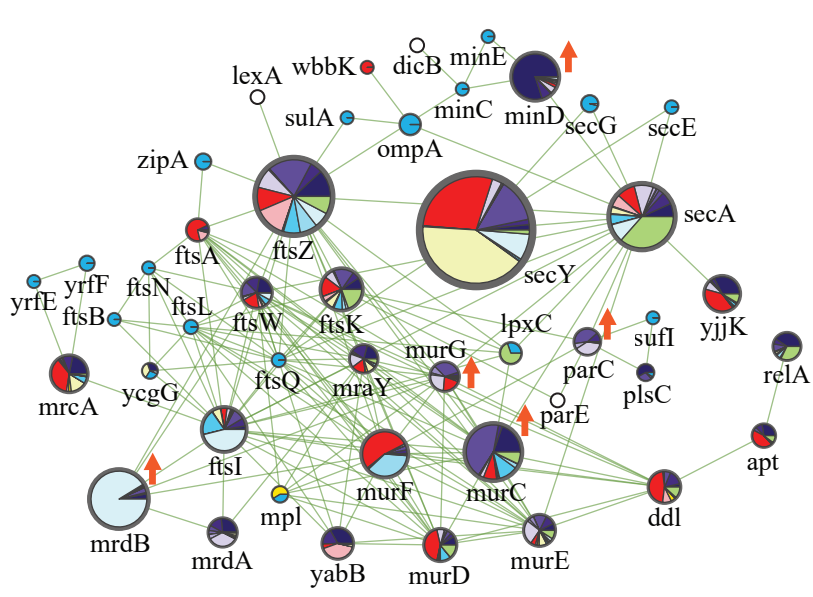

(D) Day 40 Ceca / Corn / AGPs

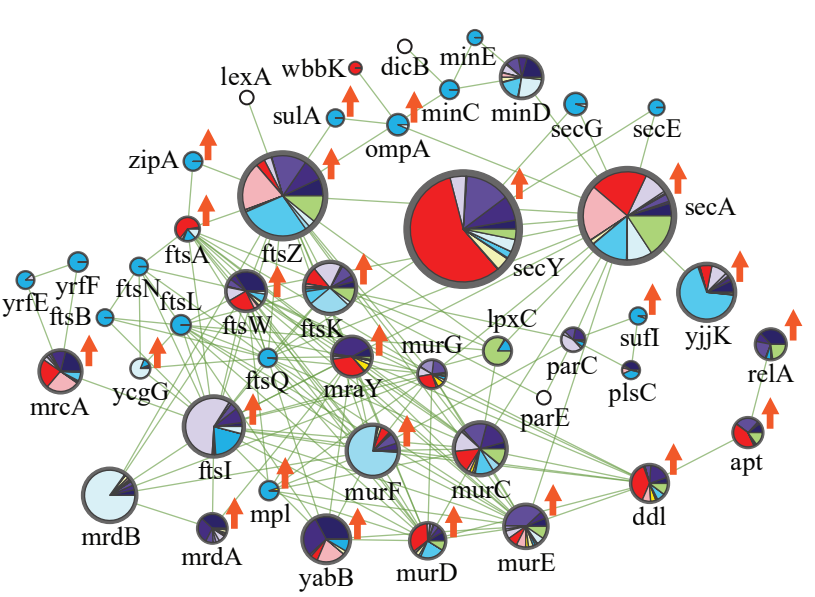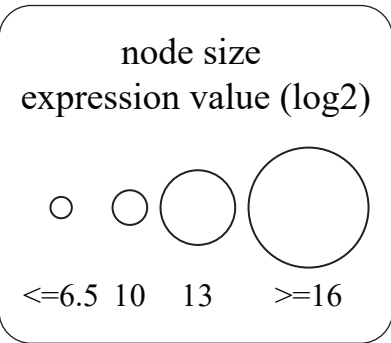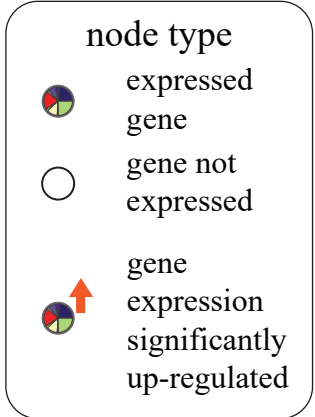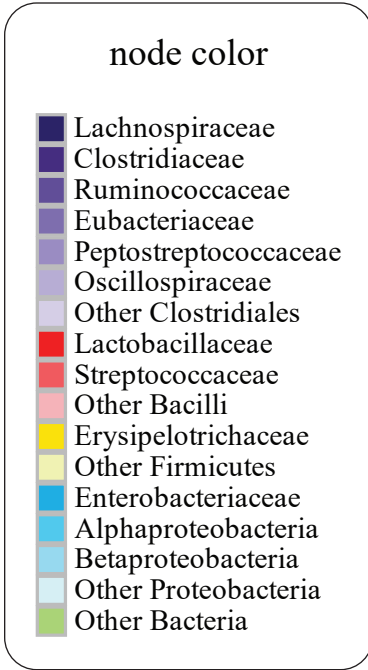

Supplement: Supplementary file 11 — Additional file 10: Supplemental Figure 10. Taxonomic contributions to gene expression profiles for proteins involved in cell wall biogenesis for ceca samples collected at day 40. Each node in the network indicates groups of orthologs corresponding to a specific E. coli gene (as indicated) involved in cell wall biogenesis. Links between nodes indicate a functional interaction as previously defined [53]. Size of the node indicates the relative expression of genes associated with each set of orthologs, with sector colours indicating the taxonomic contribution to gene expression (see key for color code). Red arrows indicate sets of orthologs that are significantly up-regulated in comparisons involving the presence/absence of AGPs. [file 40168_2022_1319_MOESM10_ESM.pdf]
