# Supplementary material for: Systematic profiling of the chicken gut microbiome reveals dietary supplementation with antibiotics alters expression of multiple microbial pathways with minimal impact on community structure
Source: Microbiome. 2022 Aug 15;10:127. doi: 10.1186/s40168-022-01319-7 (PMC9377095; doi:10.1186/s40168-022-01319-7)
Supplement: Supplementary file 3 — Additional file 2: Supplemental Figure 2. Heatmap showing normalized abundance of taxa for taxa exhibiting significant differential abundance in at least one of the comparisons shown on the right. Each column in the heatmap represents a single sample, grouped by treatment. Each row indicates an individual taxon. Columns on the right indicate significant differences in abundance across each of eight pair-wise comparisons. Taxa are ordered on the basis of magnitude of fold change across the various pairwise comparisons. [file 40168_2022_1319_MOESM2_ESM.pdf]

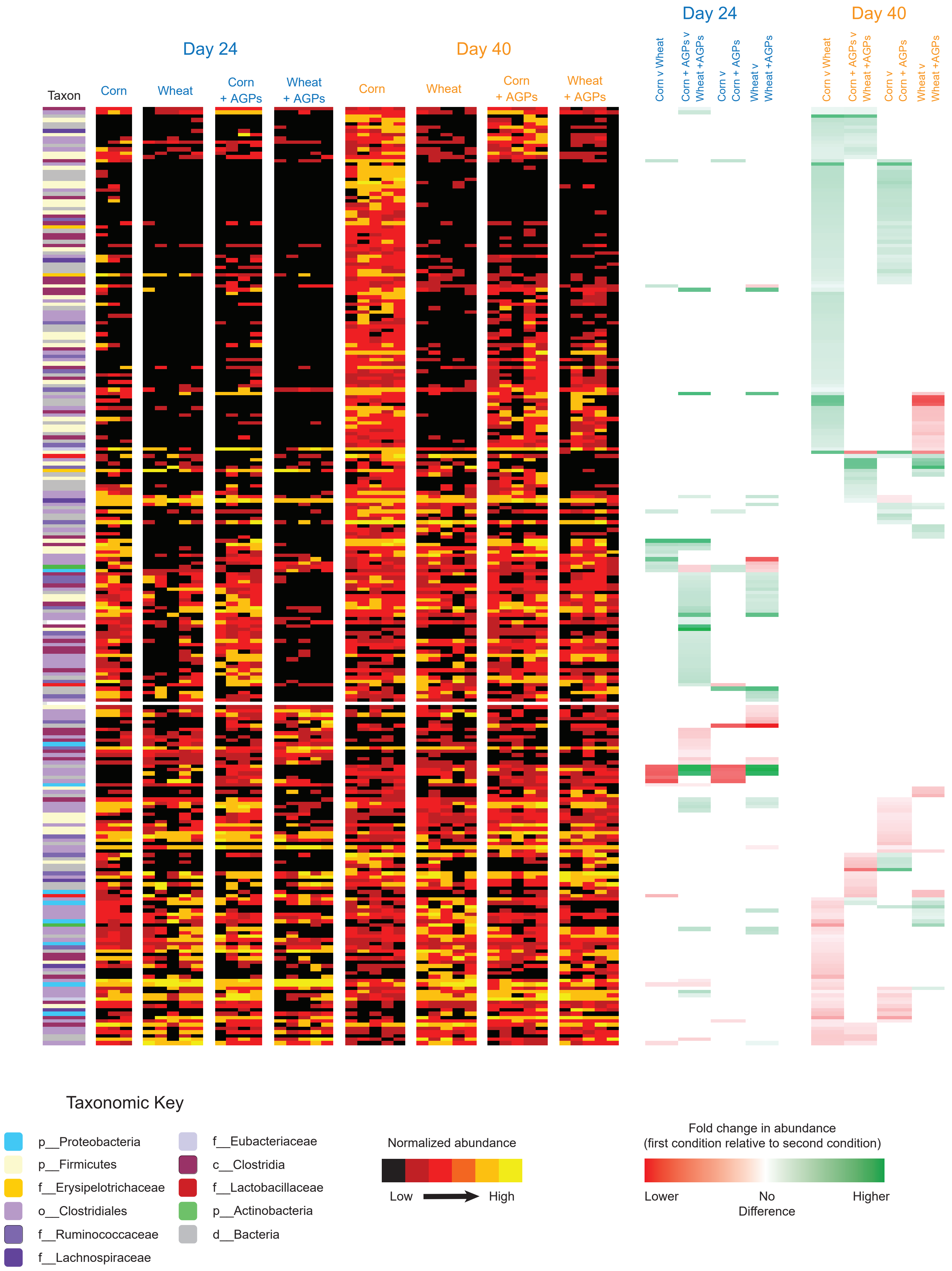

Day 24
Day 40
Taxon
Corn
Wheat
Corn + AGPs
Wheat + AGPs
Corn
Wheat
Corn + AGPs
Wheat + AGPs
Day 24
Day 40
Corn v Wheat
Corn + AGPs v Wheat +AGPs
Corn v Corn + AGPs
Wheat v Wheat +AGPs
Corn v Wheat
Corn + AGPs v Wheat +AGPs
Corn v Corn + AGPs
Wheat v Wheat +AGPs
Taxonomic Key
p__Proteobacteria
p__Firmicutes
f__Erysipelotrichaceae
o__Clostridiales
f__Ruminococcaceae
f__Lachnospiraceae
f__Eubacteriaceae
c__Clostridia
f__Lactobacillaceae
p__Actinobacteria
d__Bacteria
Normalized abundance
Low
High
Fold change in abundance
(first condition relative to second condition)
Lower
No Difference
Higher
