# Supplementary material for: Systematic profiling of the chicken gut microbiome reveals dietary supplementation with antibiotics alters expression of multiple microbial pathways with minimal impact on community structure
Source: Microbiome. 2022 Aug 15;10:127. doi: 10.1186/s40168-022-01319-7 (PMC9377095; doi:10.1186/s40168-022-01319-7)
Supplement: Supplementary file 6 — Additional file 5: Supplemental Figure 5. Expression heatmap of metabolic pathways enriched in significantly differentially expressed enzymes. There are 35 KEGG metabolic pathways enriched in significantly differentially expressed enzymes associated with samples from either ceca or jejunum. Changes of the average expression value (log2) of each gene associated with an enzyme in that pathway are indicated by red-green or blue-yellow gradients for ceca and jejunum samples, respectively. [file 40168_2022_1319_MOESM5_ESM.pdf]

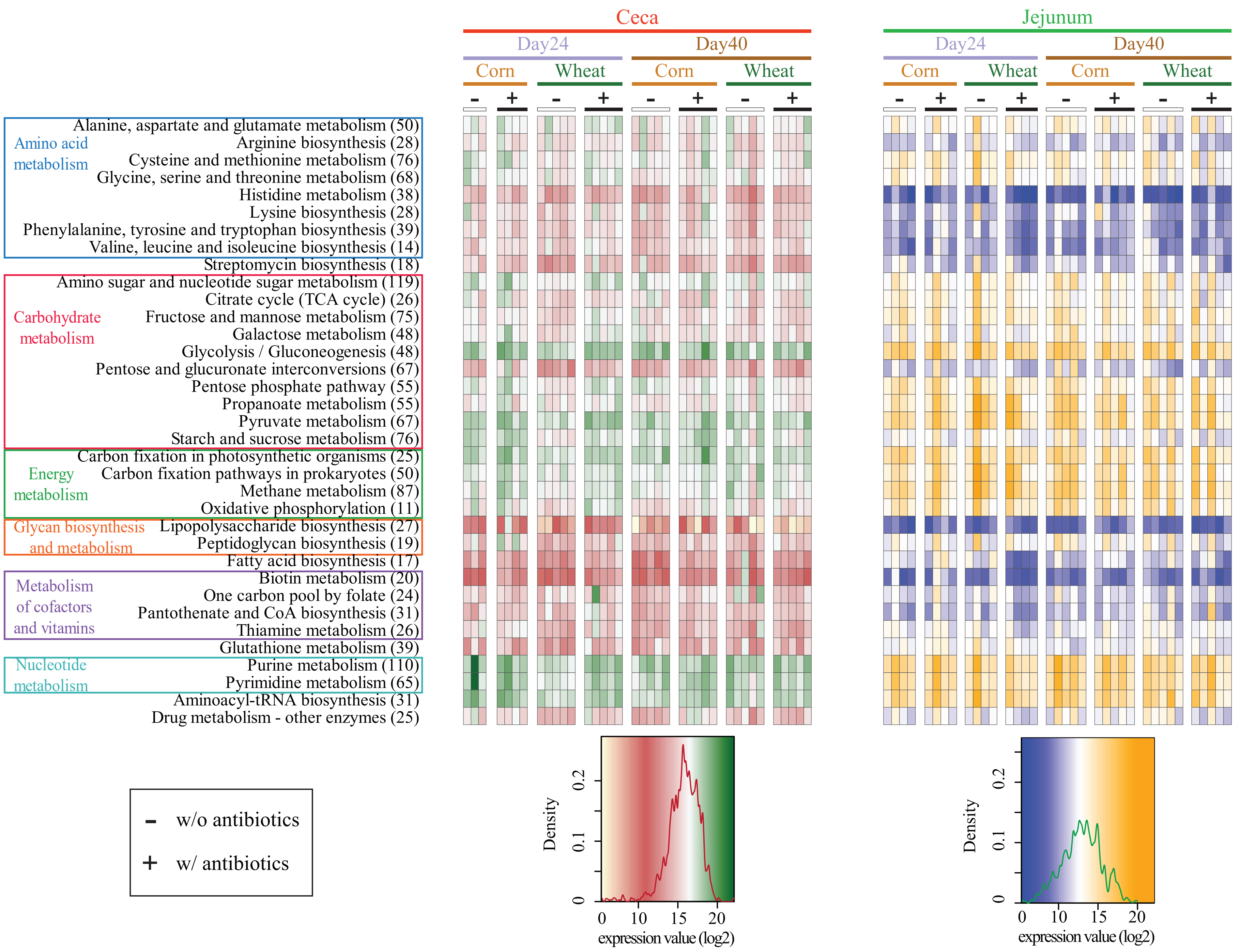

Ceca
Jejunum
Day24
Day40
Corn
Wheat
-
+
Amino acid metabolism
Alanine, aspartate and glutamate metabolism (50)
Arginine biosynthesis (28)
Cysteine and methionine metabolism (76)
Glycine, serine and threonine metabolism (68)
Histidine metabolism (38)
Lysine biosynthesis (28)
Phenylalanine, tyrosine and tryptophan biosynthesis (39)
Valine, leucine and isoleucine biosynthesis (14)
Streptomycin biosynthesis (18)
Carbohydrate metabolism
Amino sugar and nucleotide sugar metabolism (119)
Citrate cycle (TCA cycle) (26)
Fructose and mannose metabolism (75)
Galactose metabolism (48)
Glycolysis / Gluconeogenesis (48)
Pentose and glucuronate interconversions (67)
Pentose phosphate pathway (55)
Propanoate metabolism (55)
Pyruvate metabolism (67)
Starch and sucrose metabolism (76)
Energy metabolism
Carbon fixation in photosynthetic organisms (25)
Carbon fixation pathways in prokaryotes (50)
Methane metabolism (87)
Oxidative phosphorylation (11)
Glycan biosynthesis and metabolism
Lipopolysaccharide biosynthesis (27)
Peptidoglycan biosynthesis (19)
Fatty acid biosynthesis (17)
Metabolism of cofactors and vitamins
Biotin metabolism (20)
One carbon pool by folate (24)
Pantothenate and CoA biosynthesis (31)
Thiamine metabolism (26)
Glutathione metabolism (39)
Nucleotide metabolism
Purine metabolism (110)
Pyrimidine metabolism (65)
Aminoacyl-tRNA biosynthesis (31)
Drug metabolism - other enzymes (25)
- w/o antibiotics
+ w/ antibiotics
Density
0 0.1 0.2
0 10 15 20
expression value (log2)
