# Supplementary material for: Systematic profiling of the chicken gut microbiome reveals dietary supplementation with antibiotics alters expression of multiple microbial pathways with minimal impact on community structure
Source: Microbiome. 2022 Aug 15;10:127. doi: 10.1186/s40168-022-01319-7 (PMC9377095; doi:10.1186/s40168-022-01319-7)
Supplement: Supplementary file 7 — Additional file 6: Supplemental Figure 6. Taxonomic contributions to expressed enzymes in metabolic pathways across different AGP treatments and diets with Day40 ceca samples. Glycolysis/Gluconeogenesis, Pentose Phosphate and Tricarboxylic acid (TCA) cycle pathways are shown here integrated with data generated from cecal samples collected at day 40. Each pie chart represents the taxonomic distributions of an enzyme (see key for color code). The size of pie charts indicates the average expression value (with log2 transform) of genes encoding that enzyme. Pie charts with red arrows refer to enzymes that are significantly up-regulated relative to the paired (+/- AGPs) sample. The abbreviations used here can be found in Supplemental Table 11. [file 40168_2022_1319_MOESM6_ESM.pdf]

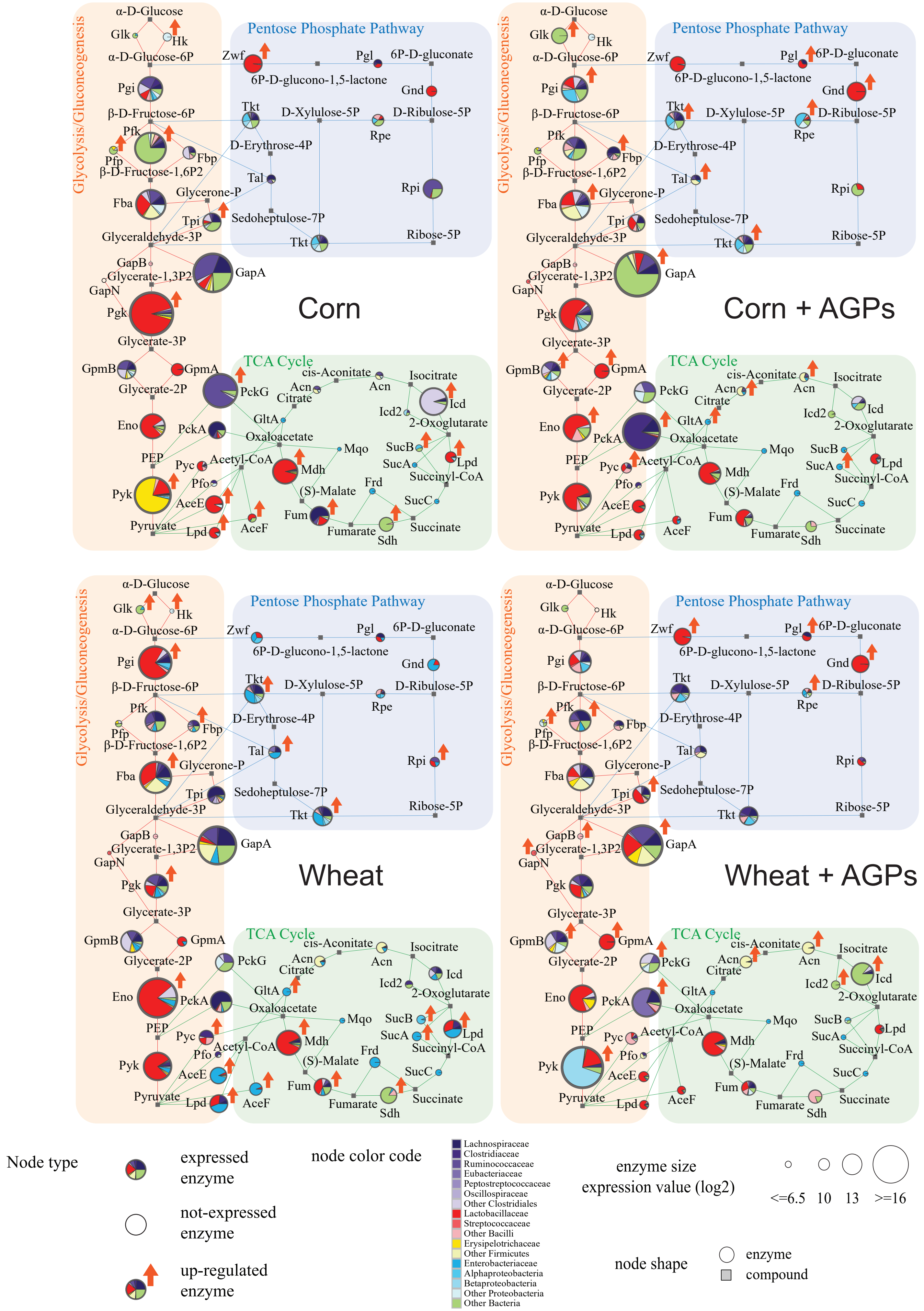
Corn
Corn + AGPs
Wheat
Wheat + AGPs
Glycolysis/Gluconeogenesis
Pentose Phosphate Pathway
TCA Cycle
α-D-Glucose
α-D-Glucose-6P
β-D-Fructose-6P
β-D-Fructose-1,6P2
Glycerone-P
Glyceraldehyde-3P
Glycerate-1,3P2
Glycerate-3P
Glycerate-2P
PEP
Pyruvate
Acetyl-CoA
6P-D-glucono-1,5-lactone
6P-D-gluconate
D-Xylulose-5P
D-Ribulose-5P
D-Erythrose-4P
Sedoheptulose-7P
Ribose-5P
cis-Aconitate
Isocitrate
Citrate
2-Oxoglutarate
Oxaloacetate
Succinyl-CoA
(S)-Malate
Fumarate
Succinate
Glk
Hk
Pgi
Pfk
Pfp
Fbp
Fba
Tpi
GapA
GapB
GapN
Pgk
GpmA
GpmB
Eno
Pyk
PckA
PckG
Pyc
Pfo
AceE
AceF
Lpd
Zwf
Pgl
Gnd
Tkt
Tal
Rpe
Rpi
GltA
Acn
Icd
Icd2
SucA
SucB
SucC
Sdh
Fum
Frd
Mdh
Mqo
Node type
expressed enzyme
not-expressed enzyme
up-regulated enzyme
node color code
Lachnospiraceae
Clostridiaceae
Ruminococcaceae
Eubacteriaceae
Peptostreptococcaceae
Oscillospiraceae
Other Clostridiales
Lactobacillaceae
Streptococcaceae
Other Bacilli
Erysipelotrichaceae
Other Firmicutes
Enterobacteriaceae
Alphaproteobacteria
Betaproteobacteria
Other Proteobacteria
Other Bacteria
enzyme size
expression value (log2)
<=6.5
10
13
>=16
node shape
enzyme
compound
