# Supplementary material for: Systematic profiling of the chicken gut microbiome reveals dietary supplementation with antibiotics alters expression of multiple microbial pathways with minimal impact on community structure
Source: Microbiome. 2022 Aug 15;10:127. doi: 10.1186/s40168-022-01319-7 (PMC9377095; doi:10.1186/s40168-022-01319-7)
Supplement: Supplementary file 12 — Additional file 11: Supplemental Figure 11. Taxonomic contributions to gene expression profiles for proteins involved in cell wall biogenesis for jejunum samples collected at day 40. Each node in the network indicates groups of orthologs corresponding to a specific E. coli gene (as indicated) involved in cell wall biogenesis. Links between nodes indicate a functional interaction as previously defined [53]. Size of the node indicates the relative expression of genes associated with each set of orthologs, with sector colours indicating the taxonomic contribution to gene expression (see key for color code). Red arrows indicate sets of orthologs that are significantly up-regulated in comparisons involving the presence/absence of AGPs. [file 40168_2022_1319_MOESM11_ESM.pdf]

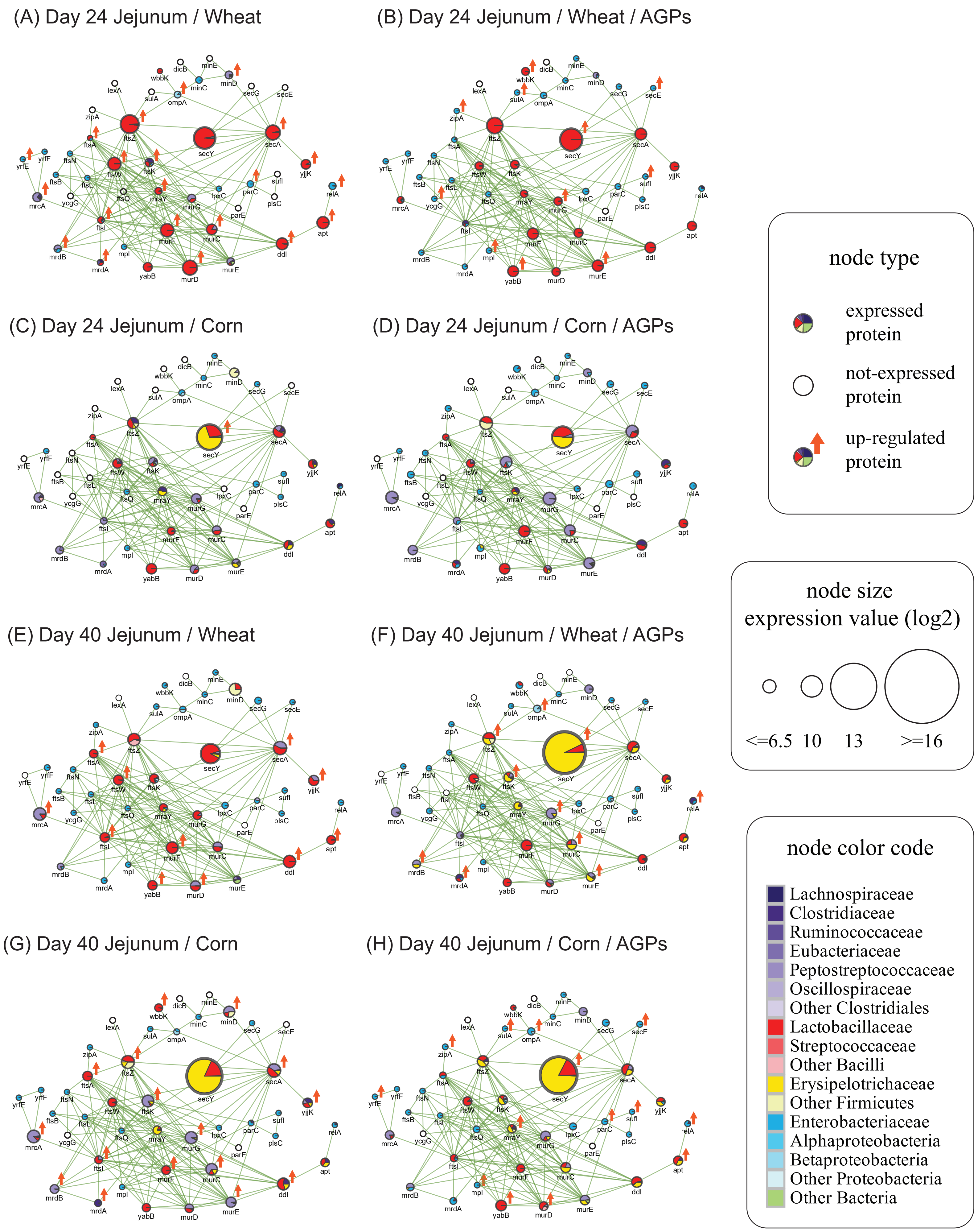
(A) Day 24 Jejunum / Wheat
(B) Day 24 Jejunum / Wheat / AGPs
(C) Day 24 Jejunum / Corn
(D) Day 24 Jejunum / Corn / AGPs
(E) Day 40 Jejunum / Wheat
(F) Day 40 Jejunum / Wheat / AGPs
(G) Day 40 Jejunum / Corn
(H) Day 40 Jejunum / Corn / AGPs
node type
expressed protein
not-expressed protein
up-regulated protein
node size
expression value (log2)
<=6.5
10
13
>=16
node color code
Lachnospiraceae
Clostridiaceae
Ruminococcaceae
Eubacteriaceae
Peptostreptococcaceae
Oscillospiraceae
Other Clostridiales
Lactobacillaceae
Streptococcaceae
Other Bacilli
Erysipelotrichaceae
Other Firmicutes
Enterobacteriaceae
Alphaproteobacteria
Betaproteobacteria
Other Proteobacteria
Other Bacteria
