# Supplementary material for: Systematic profiling of the chicken gut microbiome reveals dietary supplementation with antibiotics alters expression of multiple microbial pathways with minimal impact on community structure
Source: Microbiome. 2022 Aug 15;10:127. doi: 10.1186/s40168-022-01319-7 (PMC9377095; doi:10.1186/s40168-022-01319-7)
Supplement: Supplementary file 13 — Additional file 12: Supplemental Figure 12. Comparison of changes in pathway-specific gene expression, with changes in taxon abundance and global gene expression for three select pathways. For each pathway (glycolysis, purine metabolism and cell wall biogenesis) we calculate the shift in expression across the four conditions tested (i.e. corn + AGPs v corn; wheat + AGPs v wheat; corn v wheat; and corn + AGPs v wheat + AGPs). The absolute deviation of these changes in pathway-specific expression were then calculated relative to: 1) the relative change in abundance of that taxon (as measured by the log2 fold-change associated with that taxon according to 16S rDNA-based relative abundance); and 2) the total change in RNA for that taxon (as measured by the log2 fold-change in total taxon RNA, calculated by summing RPKM values for all genes for that taxon). Here OTUs are grouped into genera. In the box-plots, taxa with values close to zero, indicate that the shift in pathway gene expression was driven by a general shift in either taxon abundance (16S) and/or global taxon expression. Of the 48 comparisons presented, 42 show that at least 60% of taxa with pathway expression, deviate by greater than a log2 fold change from changes in relative abundance (as measured by 16S rDNA) or global taxon expression (as measured by total taxon RPKM). Note, due to lack of phylogenetic resolution associated with the 16S rDNA datasets, these comparisons feature fewer taxa than the comparisons involving taxon-specific global gene-expression. Single sample Wilcoxon tests further reveal that for 43 of the datasets, pathway-specific gene-expression significantly deviates from either taxon-abundance or global gene expression (* p<0.05; ** p< 0.01; *** p<0.001). The lower panels show the 20 genera exhibiting the greatest divergence in pathway expression relative to global gene expression. For the Corn v Corn+AGP comparison for the glycolysis pathway, we identified ‘Other Bacteria’ and various P [file 40168_2022_1319_MOESM12_ESM.pdf]

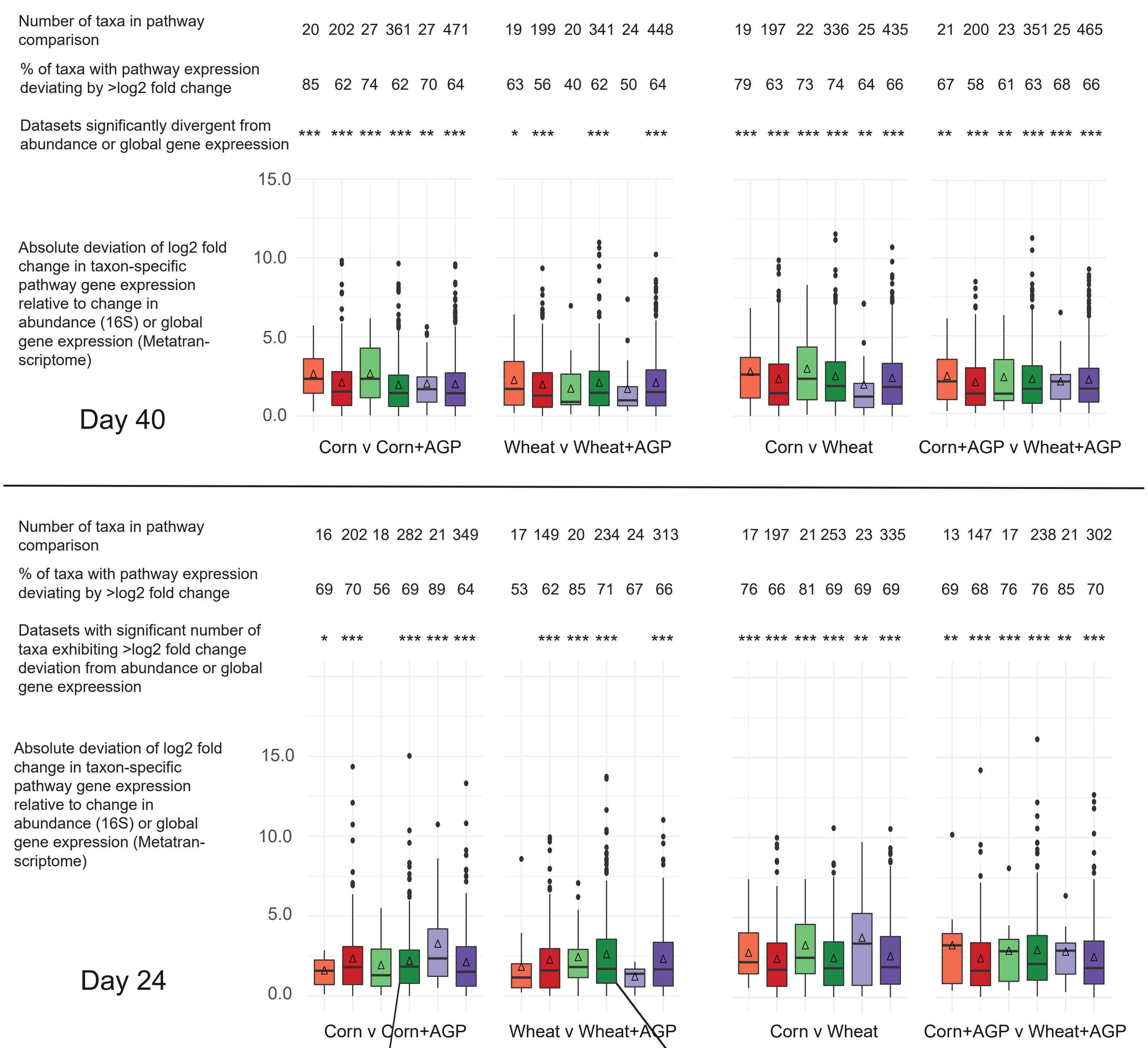

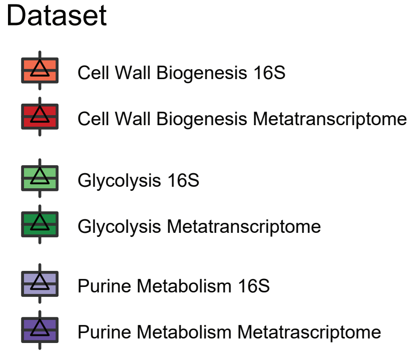

Top 20 Taxa

Genus	Clade	log2FC pathway expression	log2FC all gene expression	Deviation between pathway and all gene expression
Providencia	Other Proteobacteria	4.01	-5.58	9.59
Lacunisphaera	Other Bacteria	9.57	1.23	8.34
Leclercia	Enterobacteriaceae	0.38	-7.26	7.64
Cellulomonas	Other Bacteria	8.54	1.20	7.34
Chromohalobacter	Other Proteobacteria	1.94	-4.59	6.53
Jeongeupia	Betaproteobacteria	4.48	-1.73	6.21
Acetobacter	Alphaproteobacteria	1.85	-3.84	5.69
Salmonella	Enterobacteriaceae	-2.17	-7.70	5.53
Bifidobacterium	Other Bacteria	3.44	-0.75	4.19
Chlamydia	Other Bacteria	3.68	-0.47	4.15
Erwinia	Other Proteobacteria	0.22	-3.90	4.12
Megasphaera	Other Firmicutes	2.98	-1.08	4.06
Elusimicrobium	Other Bacteria	3.66	-0.33	3.98
Brachyspira	Other Bacteria	2.57	-1.34	3.91
Synechococcus	Other Bacteria	0.28	-3.53	3.82
Roseomonas	Alphaproteobacteria	4.07	0.45	3.62
Novibacillus	Other Bacilli	3.56	0.00	3.56
Xylanimonas	Other Bacteria	2.56	-0.96	3.52
Rummeliibacillus	Other Bacilli	0.26	-3.07	3.33
Macrococcus	Other Bacilli	2.14	-1.09	3.23

Top 20 Taxa

Genus	Clade	log2FC pathway expression	log2FC all gene expression	Deviation between pathway and all gene expression
Thermosynechococcus	Other Bacteria	5.18	-6.47	11.64
Mycobacterium	Other Bacteria	8.67	-1.73	10.41
Rhodanobacter	Other Proteobacteria	4.10	-5.21	9.31
Eggerthella	Other Bacteria	7.36	-1.61	8.97
Filifactor	Peptostreptococcaceae	9.66	0.78	8.88
Kocuria	Other Bacteria	-0.21	-8.14	7.93
Calothrix	Other Bacteria	0.72	-7.10	7.82
Arsenicicoccus	Other Bacteria	4.59	-1.57	6.16
Sulfurihydrogenibium	Other Bacteria	5.27	-0.85	6.12
Jeongeupia	Betaproteobacteria	7.00	0.96	6.03
Gemmiger	Other Firmicutes	1.88	-4.13	6.02
Pseudomonas	Other Proteobacteria	2.16	-3.48	5.64
Corallococcus	Other Proteobacteria	5.16	-0.42	5.58
Aeribacillus	Other Bacilli	3.62	-1.93	5.55
Moorella	Other Firmicutes	2.16	-3.38	5.54
Defluviitoga	Other Bacteria	2.56	-2.62	5.18
Pelosinus	Other Firmicutes	4.31	-0.65	4.96
Geosporobacter	Clostridiaceae	3.76	-1.02	4.79
Cystobacter	Other Proteobacteria	3.50	-1.20	4.70
Muribaculum	Other Bacteria	-2.26	-6.66	4.40
