# Supplementary material for: Systematic profiling of the chicken gut microbiome reveals dietary supplementation with antibiotics alters expression of multiple microbial pathways with minimal impact on community structure
Source: Microbiome. 2022 Aug 15;10:127. doi: 10.1186/s40168-022-01319-7 (PMC9377095; doi:10.1186/s40168-022-01319-7)
Supplement: Supplementary file 14 — Additional file 13: Supplemental Figure 13. Taxon-specific expression profiles for genes encoding proteins involved in cell wall biogenesis for ceca samples collected at day 24. Each network represents proteins and their interactions associated with the three select taxa. Nodes represent an ortholog of an E. coli protein previously predicted to be involved in cell wall biogenesis [53]. Size of the node indicates the relative expression of genes associated with the ortholog for that taxon. Links between nodes indicate a functional interaction as defined by the STRING protein interaction database [75], with those in green also found in the previously described E. coli network, and those in orange, not found in the previously described E. coli network. [file 40168_2022_1319_MOESM13_ESM.pdf]

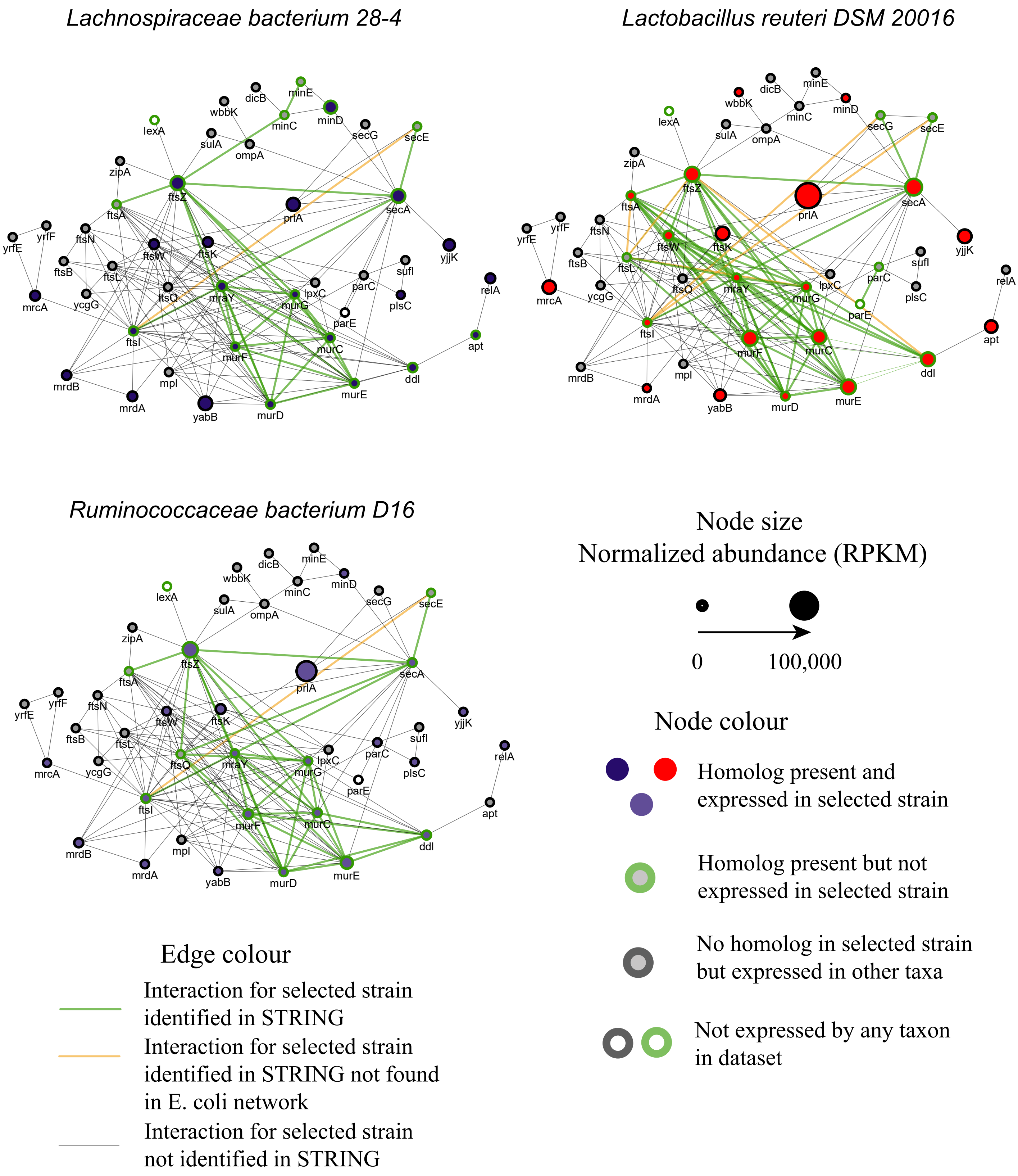

Lachnospiraceae bacterium 28-4
minE
dicB
wbbK
minC
minD
lexA
sulA
ompA
secG
secE
zipA
ftsZ
secA
prlA
ftsA
yrfF
ftsN
yrfE
ftsW
ftsK
yjjK
ftsB
ftsL
sufI
parC
relA
mrcA
ycgG
ftsQ
mraY
lpxC
murG
plsC
parE
ftsI
murF
murC
apt
mrdB
mpl
ddl
mrdA
yabB
murD
murE
Lactobacillus reuteri DSM 20016
dicB
minE
wbbK
minD
minC
lexA
sulA
ompA
secG
secE
zipA
ftsZ
prlA
secA
ftsA
yrfF
yrfE
ftsN
ftsW
ftsK
yjjK
ftsB
ftsL
sufI
relA
ftsQ
lpxC
parC
mrcA
ycgG
mraY
murG
plsC
parE
ftsI
murF
murC
apt
mpl
mrdB
ddl
mrdA
yabB
murD
murE
Ruminococcaceae bacterium D16
minE
dicB
wbbK
minD
minC
lexA
secG
sulA
ompA
secE
zipA
ftsZ
prlA
secA
ftsA
yrfF
ftsN
yrfE
ftsW
ftsK
yjjK
ftsB
ftsL
sufI
relA
parC
mrcA
ycgG
ftsQ
mraY
lpxC
murG
plsC
parE
apt
ftsI
murF
murC
mrdB
ddl
mpl
mrdA
yabB
murD
murE
Node size
Normalized abundance (RPKM)
0
100,000
Node colour
Homolog present and expressed in selected strain
Homolog present but not expressed in selected strain
No homolog in selected strain but expressed in other taxa
Not expressed by any taxon in dataset
Edge colour
Interaction for selected strain identified in STRING
Interaction for selected strain identified in STRING not found in E. coli network
Interaction for selected strain not identified in STRING
