# Supplementary material for: Systematic profiling of the chicken gut microbiome reveals dietary supplementation with antibiotics alters expression of multiple microbial pathways with minimal impact on community structure
Source: Microbiome. 2022 Aug 15;10:127. doi: 10.1186/s40168-022-01319-7 (PMC9377095; doi:10.1186/s40168-022-01319-7)
Supplement: Supplementary file 17 — Additional file 16: Supplemental Table 3. Shannon diversity indices of samples classified according to age, site, and treatment. Averages of each grouping are shown with standard deviation in brackets. [file 40168_2022_1319_MOESM16_ESM.docx]

|  |  | Corn and antibiotics | Corn | Wheat and antibiotics | Wheat |
| --- | --- | --- | --- | --- | --- |
| Gizzard | Day 10 | 2.53 (0.17) | 2.31 (0.42) | 2.47 (0.21) | 2.78 (0.07) |
|  | Day 24 | 3.01 (0.65) | 2.29 (0.61) | 2.82 (0.3) | 3.74 (1.07) |
|  | Day 40 | 2.67 (0.55) | **2.36 (0.2)^a^** | 2.83 (0.24) | **2.95 (0.25)^a^** |
|  |  |  |  |  |  |
| Duodenum | Day 10 | 4.55 (0.92) | 3.23 (1.31) | 3.62 (0.84) | 2.49 (0.17) |
|  | Day 24 | 3.74 (0.83) | 3.4 (0.47) | 2.57 (0.97) | 3.93 (1.26) |
|  | Day 40 | **4.56 (0.56)^b,c^** | **3.16 (0.36)^b^** | **3.39 (0.53)^c^** | 3.07 (0.51) |
|  |  |  |  |  |  |
| Jejunum | Day 10 | **3.29 (0.4)^d^** | **3.37 (0.47)^e^** | **2.14 (0.25)^d,f^** | **2.7 (0.15)^e,f^** |
|  | Day 24 | 2.59 (0.85) | 1.97 (0.8) | 1.44 (0.38) | 2.29 (1.63) |
|  | Day 40 | 1.97 (0.21) | 2.14 (0.53) | 2.80 (1.45) | 2.30 (1.10) |
|  |  |  |  |  |  |
| Ileum | Day 10 | 3.46 (0.99) | 2.85 (0.31) | 2.37 (0.41) | 2.44 (0.33) |
|  | Day 24 | 1.96 (0.42) | 1.85 (0.36) | 2.03 (0.64) | 2.28 (0.25) |
|  | Day 40 | 2.65 (0.65) | 2.46 (0.77) | 3.1 (0.44) | 3.1 (0.7) |
|  |  |  |  |  |  |
| Cecum | Day 10 | 3.83 (0.12) | 4.56 (0.57) | 3.68 (0.54) | 3.28 (0.88) |
|  | Day 24 | 5.78 (0.38) | **6.08 (0.26)^g^** | **4.14 (0.21)^h^** | **5.03 (0.49)^g,h^** |
|  | Day 40 | 6.29 (0.45) | **6.9 (0.27)^i^** | 5.72 (0.72) | **5.74 (0.37)^i^** |
|  |  |  |  |  |  |
| Colon | Day 10 | 4.17 (0.58) | 4.16 (0.8) | 4.31 (0.55) | 3.63 (0.34) |
|  | Day 24 | 5.28 (1.23) | 4.84 (1.61) | 3.67 (1.03) | 4.81 (0.48) |
|  | Day 40 | 6.21 (0.31) | 6.32 (1.26) | 5.36 (0.61) | 5.02 (0.86) |

**Supplemental Table 3. Shannon diversity indices of samples classified according to age, site, and treatment.** Averages of each grouping are shown with standard deviation in brackets.

**^a-j^** Samples with matching superscripts exhibit significant differences in diversity
